# An Integrative Network Approach to Identify Common Genes for the Therapeutics in Tuberculosis and Its Overlapping Non-Communicable Diseases

**DOI:** 10.3389/fphar.2021.770762

**Published:** 2022-01-27

**Authors:** Aftab Alam, Hala Abubaker Bagabir, Armiya Sultan, Mohd Faizan Siddiqui, Nikhat Imam, Mustfa F Alkhanani, Ahmad Alsulimani, Shafiul Haque, Romana Ishrat

**Affiliations:** ^1^ Centre for Interdisciplinary Research in Basic Sciences, Jamia Millia Islamia, New Delhi, India; ^2^ Department of Physiology, Faculty of Medicine, King Abdulaziz University, Rabigh, Saudi Arabia; ^3^ Department of Biosciences, Jamia Millia Islamia, New Delhi, India; ^4^ International Medical Faculty, Osh State University, Osh, Kyrgyzstan; ^5^ Department of Mathematics, Institute of Computer Science and Information Technology, Magadh University, Bodh Gaya, India; ^6^ Emergency Service Department, College of Applied Sciences, AlMaarefa University, Riyadh, Saudi Arabia; ^7^ Medical Laboratory Technology Department, College of Applied Medical Sciences, Jazan University, Jazan, Saudi Arabia; ^8^ Research and Scientific Studies Unit, College of Nursing and Allied Health Sciences, Jazan University, Jazan, Saudi Arabia

**Keywords:** Network Biology, Network Medicines, Disease-disease relationship, Disease-target interaction, MTB and NCDs

## Abstract

Tuberculosis (TB) is the leading cause of death from a single infectious agent. The estimated total global TB deaths in 2019 were 1.4 million. The decline in TB incidence rate is very slow, while the burden of noncommunicable diseases (NCDs) is exponentially increasing in low- and middle-income countries, where the prevention and treatment of TB disease remains a great burden, and there is enough empirical evidence (scientific evidence) to justify a greater research emphasis on the syndemic interaction between TB and NCDs. The current study was proposed to build a disease-gene network based on overlapping TB with NCDs (overlapping means genes involved in TB and other/s NCDs), *such as* Parkinson’s disease, cardiovascular disease, diabetes mellitus, rheumatoid arthritis, and lung cancer. We compared the TB-associated genes with genes of its overlapping NCDs to determine the gene-disease relationship. Next, we constructed the gene interaction network of disease-genes by integrating curated and experimentally validated interactions in humans and find the 13 highly clustered modules in the network, which contains a total of 86 hub genes that are commonly associated with TB and its overlapping NCDs, which are largely involved in the Inflammatory response, cellular response to cytokine stimulus, response to cytokine, cytokine-mediated signaling pathway, defense response, response to stress and immune system process. Moreover, the identified hub genes and their respective drugs were exploited to build a bipartite network that assists in deciphering the drug-target interaction, highlighting the influential roles of these drugs on apparently unrelated targets and pathways. Targeting these hub proteins by using drugs combination or drug repurposing approaches will improve the clinical conditions in comorbidity, enhance the potency of a few drugs, and give a synergistic effect with better outcomes. Thus, understanding the *Mycobacterium tuberculosis* (Mtb) infection and associated NCDs is a high priority to contain its short and long-term effects on human health. Our network-based analysis opens a new horizon for more personalized treatment, drug-repurposing opportunities, investigates new targets, multidrug treatment, and can uncover several side effects of unrelated drugs for TB and its overlapping NCDs.

## Introduction

Tuberculosis (TB), a communicable disease caused by *bacillus Mycobacterium tuberculosis*, is the leading cause of death from a single infectious agent. Globally, an estimated 10.0 million people developed tuberculosis in 2020 (WHO Global Tuberculosis Report-2021). Among these cases, 56% of individuals were men aged ≥15 years, 32% were women, and 12% were children aged <15 years. Most affected people were from the region of South-East Asia (44%), Africa (25%), and the Western Pacific (18%). A total of 1.5 million people died from TB in 2020 (including 214,000 people with HIV). Worldwide, TB is the 13th leading cause of death and the second leading infectious killer after COVID-19 (above HIV/AIDS).

TB is still considered a deadly disease, particularly in high TB burden countries like India, China, Indonesia, Philippines, Pakistan, Nigeria, Bangladesh, and South Africa (Bhatia et al., 2020). WHO reports reflect that the TB incidence rate decline is very slow, while the burden of noncommunicable diseases (NCDs) is exponentially increasing worldwide (WHO Global Tuberculosis Report, 2020; WHO Noncommunicable Diseases Progress Monitor, 2020).

In the long term, tuberculosis may lead to collapse in immune surveillance, enhancing one’s susceptibility to non-communicable diseases (NCDs), which together contribute to two-thirds of the worldwide mortality (Marais et al., 2013; Peltzer, 2018). Emerging empirical evidence justifies the convergence of TB with NCDs such as Parkinson’s disease (PD) (Shen et al., 2016), cardiovascular diseases (CVD) (Huaman et al., 2015), diabetes mellitus (DM) (Menon et al., 2016), rheumatoid arthritis (RA) (Carmona et al., 2003), and lung cancer (LC) (Chai and Shi, 2020).

Many Infectious diseases have been reported to contribute to the development of PD (Harris et al., 2012; Vlajinac et al., 2013; Tan et al., 2015). Patients with TB have been reported to have a 1.38-fold higher risk of developing PD as compared to control subjects (Shen et al., 2016). The related mechanisms are not known; however, it is thought that pro-inflammatory responses generated in TB may be a key driving process associated with PD’s pathogenesis (Kaufmann and Dorhoi, 2013). In 2018, Anetta, et al. suggested that the mechanism of our immune cells (macrophages) for wipe out the TB infection might also be involved in Parkinson’s disease. Generally, mutation in *LRRK2* gene make the LRRK2-protein overactive in Parkinson’s disease. The *LRRK2* prevents phagosomes from fusing with lysosomes in macrophages, making them less efficient at clearing Mtb. Deleting the *LRRK2* gene or treating the cells with an *LRRK2* blocker significantly reduced the Mtb infection. So, drugs developed to treat PD (*LRRK2* inhibitors) might work for TB too (Härtlova et al., 2018).

Tuberculosis and NCDs may not only co-exist but also increases the risk of each other. Developing tuberculosis disease may indicate background dysregulation of immune responses (innate immunity) in susceptible hosts, as these same abnormal responses may also predispose to CVD (Marais et al., 2013; Huaman et al., 2015). The burden of both diseases is enormous across the world and augment the risk of each other. The potential mechanistic association of TB with CVD is based on persistent immune activation in TB. Antibodies to mycobacterial HSP65 cross-reacting with self-antigens in human vessels leading to autoimmunity may also affect CVD risk (Huaman et al., 2015). The convergence of both diseases is posing a greater challenge for treatment plans in overlapping TB and CVD.

The burden of diabetes has also been a major health concern in South Asian countries, with an estimated rise of more than 151% between 2,000 and 2020 (Jayawardena et al., 2012; Shrestha et al., 2020). There is a bidirectional connection between TB and DM, and their synergistic role in causing human disease is well recognized. There is very little information available about the exact mechanism of how diabetes comorbidity impacts health outcomes in TB patients. However, there is some evidence for the negative impact of diabetes comorbidity on the TB treatment outcome (Dooley et al., 2009; Wang et al., 2009; Chiang et al., 2015), specifically for delays in treatment failures, mycobacterial clearance, death, relapse, and re-infection.

Furthermore, It has been also seen that tuberculosis lead to impair the induction of glucose intolerance and worsening of glycaemic control in DM patients (Melmed, 2011). TB also has a bidirectional epidemiological association with RA and has reported that patients with RA have a 4-fold higher risk of developing TB than the control population (Carmona et al., 2003). In this double burden disease, on one side, immunological responses involving Th1 mediated activation of cytokines are key to protect against TB (Barnes and Wizel, 2000; Stenger, 2005; Yasui, 2014), while on the other side, anti-rheumatic drugs (tDMARDs) that act against the host immune system are increasing the risk of TB in RA patients (Lim et al., 2017). Moreover, several studies have reported the reactivation of TB in RA patients treated with anti–TNF-α agents (Keane et al., 2001; Ormerod, 2004; Dixon et al., 2010).

The overlapping of TB and lung cancer has attracted many researchers in the last few decades. Many studies have reported that TB is associated with cancer and increases the risk and mortality of lung cancer and vice versa (Leung et al., 2013). However, data related to TB treatment of LC patients is still incomplete and inconsistent. The connection between tuberculosis and lung cancer is still not completely understood. Lung parenchyma tissue involved in both diseases, regular cough in lung cancer, morphological vascular variations, lymphocytosis mechanisms, and production of immune system mediators like interleukins are all among the factors leading to the hypothesis about the major role of tuberculosis in lung cancer (Liang et al., 2009; Brenner et al., 2011; Bhatt et al., 2012). It has been shown that the inflammatory process is one of the potential factors of lung cancer, and the crucial inflammation-inducing factors are tuberculosis (TB), pneumonia, and chronic bronchitis, among which TB has a more profound role in the emergence of lung cancer (Keikha and Esfahani, 2018). Many studies reported that the induction of necrosis and apoptosis or TB reactivation might result in increasing TNF-α and IL-17 that will either decreases the activity of P53 or increase the BCL-2 expression, decrease Bax-T, and cause the inhibition of caspase-3 expression due to decreasing the expression of mitochondria cytochrome oxidase (Mariani et al., 2001; Liuzzo et al., 2013). It is clear that the epidemiological shift creates a double disease burden in the affected population and is rising as a critical health problem globally. The intersection between TB and other NCDs poses pharmacological issues and a great challenge for the co-management and treatment, reflecting a need for a radical shift, emphasizing common treatment targets irrespective of vertical approaches focused on individual diseases.

Recently, Gysi et al. implemented a network-medicine and drug-repurposing approach to identify repurposable drugs for COVID-19 (Morselli Gysi et al., 2021). Sakle et al. have used a network pharmacology-based approach to prove that Caesalpinia pulcherima (CP) is a multi-target herb for the betterment of clinical uses for the treatment of breast cancer (Sakle et al., 2020). Besides, Azuaje, et al. had provided systemic insights into cardiovascular effects of non-cardiovascular drugs by combining different sources of drug and protein interaction information to assemble the myocardial infarction drug-target interactome network (Azuaje et al., 2011) In another similar study, Kim et al. has suggested that network-based drug-disease proximity offers a novel perspective into a drug’s therapeutic effect in the Systemic Sclerosis (SSc) disease and that could be applied to drug combinations or drug repositioning (Kim et al., 2020).

Network analysis is uniquely suited to approach based on the theoretical paradigm and methodological tools to research, describe, explore, and understand structural and relational aspects of human health and diseases (Luke and Harris, 2007). Network-based studies are emerging as an important tool to determine the disease susceptibility genes and their relationship with different diseases. These studies have also improved our understanding of drug targets and their effects and suggested new drug targets, therapeutics, and therapeutic management approaches in severe diseases (Berger and Iyengar, 2009). Analysis of networks is significantly contributing to the genesis of systems pharmacology.

The current study was proposed to build a disease network based on the overlapping of TB with other NCDs, namely PD, CVD, DM, RA, and LC. The disease network was analyzed to identify the TB-associated genes that are commonly associated with other NCDs and determine the gene-disease relationship. Next, we constructed the gene interaction network of each disease independently by integrating curated and experimentally validated interactions in humans (Barabási and Oltvai, 2004). All the gene interaction networks were merged into a single large network using the graph union operation, and the network’s structural properties were distinguished through the behavior of the topological parameters followed by modules identification because modules in a large network are functionally and statistically significant interacting clusters of nodes that resemble community organizations. Next, we generate and analyzed the drug-target interactome network, which integrates data about clinically relevant drug-drug and drug-target interactions. The resulting network lays the basis for a broader picture of the drug-target interaction landscape. The overall study offers new opportunities for understanding the biological basis of treatment efficacy and targeted and multidrug therapy in TB and its overlapping NCDs.

## Material and Methods

**FIGURE 1:**
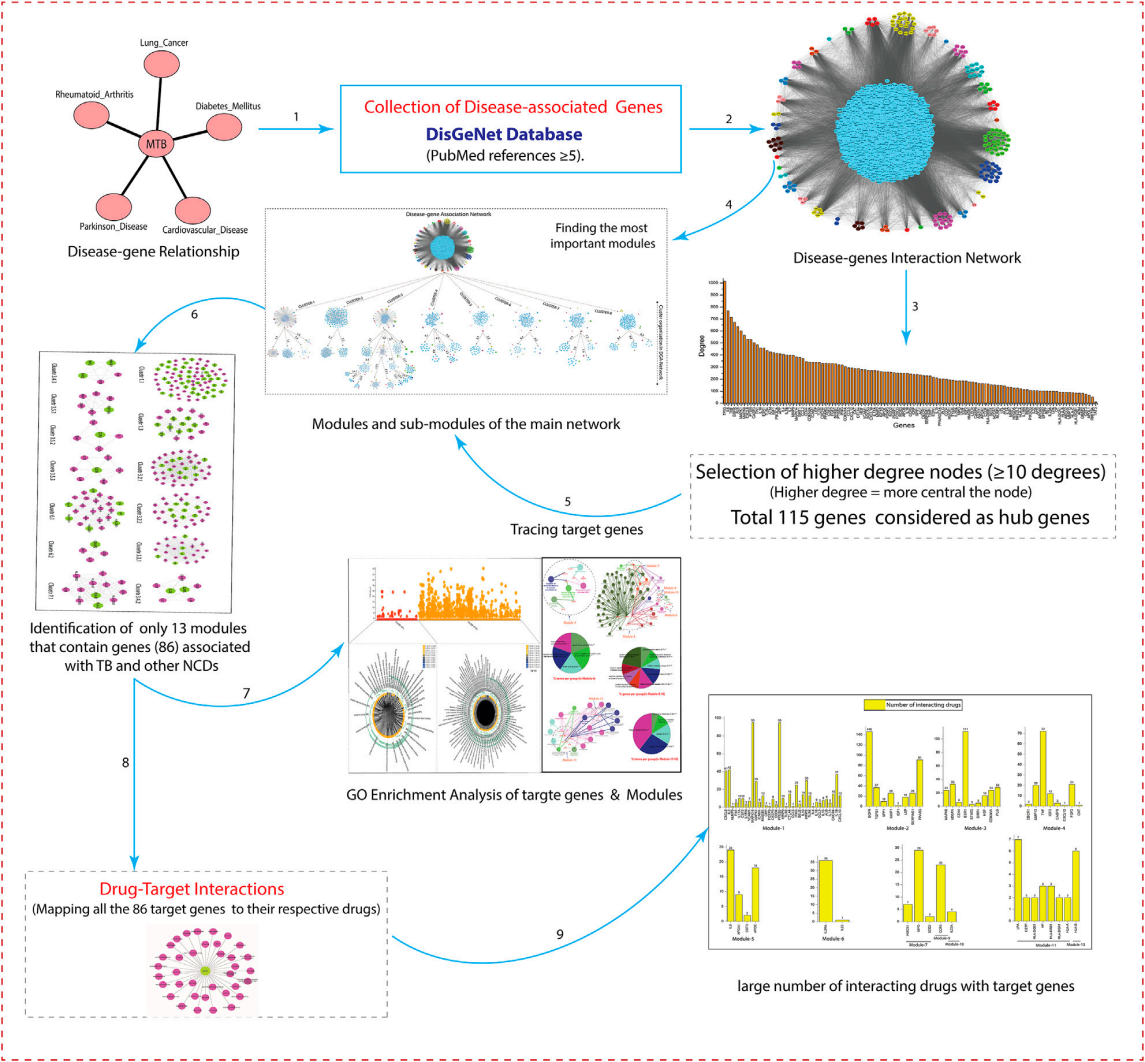
The schematic representation of workflow and methodology used in this study.

The schematic workflow of this study is represented in Figure 1.

### Collection of Disease-Associated Genes

Disease-associated genes of Tuberculosis (TB), along with its associated non-communicable diseases, namely Parkinson disease (PD), cardiovascular disease (CVD), diabetes mellitus (DM), rheumatoid arthritis (RA), and lung cancer (LC), were obtained from the DisGeNet (v7.0), a database comprehensively integrated expert-curated. DisGeNET contains a compilation of genes associated to diseases, that taken from several publicly available databases including, UniProt/SwissProt, Cancer Genome Interpreter (CGI), Comparative Toxicogenomics Database™ (CTD™), Orphanet, Mouse Genome Database (MGD), PsyGeNET, Genomics England, ClinGen, and Rat Genome Database (RGD) (Piñero et al., 2017).

The gene-disease correlation was analyzed and selected only those genes with many publications supporting the association (PubMed references ≥5). Genes may be associated with one or more than one disease or may be linked with the convergence of one disease with another. Further, to determine the gene-disease relationship, an online tool from *Bioinformatics and Evolutionary Genomics* lab (http://bioinformatics.psb.ugent.be/webtools/Venn/) was used to compare the tuberculosis-associated genes with its overlapping NCDs (PD, CVD, DM, RA, and LC).

### Construction of Gene Interaction Network of Disease-Associated Genes

The gene interactions network of each disease was built independently by integrating curated and experimentally validated interactions in humans from the IntAct (Aranda et al., 2010), BioGrid (Oughtred et al., 2019), Mentha (Calderone et al., 2013), Reactome-FIs(Croft et al., 2011), InnateDB-All (Piñero et al., 2017), and MINT (Ceol et al., 2010) databases. All these databases provide freely accessible open-source databases and analysis tools for molecular interaction data. All the networks were visualized in Cytoscape-6.1 (Shannon et al., 2003). After visualization, all the networks were merged into a single large network using the graph union operation. The duplicate edges and self-loops were removed. In the network, each node represents the gene, and edges represent the connection between the nodes.

Moreover, network analyzers were employed to calculate basic network properties, The complex network’s structural properties were distinguished through the behavior of the topological parameters. It helps to understand the network structure, which facilitates understanding the hidden mechanisms (Alam et al., 2019). The following networks properties were analyzed to seek the important behaviours of the network:

• *Degree distribution:* In a biological network, the degree(k) of node(n) is the total number of connections with other nodes. The probability distribution of this degree is called degree distribution (P(k)).

$$P\left(k\right)=\frac{n_{k}}{N}$$ (1)

Where **n**_**k**_ = No. of nodes with degree k**.** **N**= The total number of nodes in the network.

• *Neighborhood connectivity:* It gives the average of the neighborhood connectivity of all the nodes (N) with the number of neighbors. So *neighborhood connectivity [C*_*N*_ (*K*)] *is:*

$$C_{N}\left(k\right)=\sum_{q}\mathrm{qP}\left(\frac{q}{k}\right)$$ (2)

Where, $P\;\left(\frac{q}{k}\right)$ = The conditional probability.

• *Clustering coefficient:* The ratio of a number of edges (**e**_**i**_) between the node’s neighbor or the highest numbers of edges that could cause possibly occurrence among the nodes. So, the total network cluster coefficient is the average cluster coefficient of all nodes (**i**th) in the network.

$$C\left(k_{i}\right)=\frac{2e_{i}}{k_{i}\left(k_{i}-1\right)}$$ (3)

• *Betweenness centrality:* A node’s betweenness centrality shows the importance of information flow from one node to another via the shortest path. From node (**i**) to node (**j)**, the geodesic paths are shown by ‘**dij(v)**’, which passing via node ‘**v**’ and ‘**dij**’.

$$C_{B}\left(v\right)=\;\sum_{i,j,i\neq j\neq k}\frac{d_{\mathrm{ij}}\left(v\right)}{d_{\mathrm{ij}}}$$ (4)

• *Closeness centrality:* In the network, how quickly information is passing from one node (i) to another (j) is calculated by Closeness centrality (C_C_).

$$C_{C}\left(i\right)=\frac{n}{\sum_{j}d_{\mathrm{ij}}}$$ (5)

• *Eigenvector centrality:* The eigenvector centrality of a node “**i** (**C**_**E**_(**i))**” is proportionate to the total of **i**’s neighbor centralities.

$$C_{E}\left(i\right)=\frac{1}{\lambda }\sum_{j=\mathrm{nn}\left(i\right)}v_{j}$$ (6)

Where, **nn(i)** = Closest neighbours of nodes (“**i**”).

**λ** = Eigenvalue of the eigenvector.
**v**_**i**_ = ‘**Av**_**i**_ **= λv**_**i**_’ where, ‘**A**’ (adjacency matrix).

### Finding the Most Important Modules/sub-modules

We used MCODE to find the most important modules in the network. Here “important” means highly interconnected, or dense regions of the network that represent modules that act in concert to perform specific biological functions. The MCODE is a novel graph-theoretic clustering algorithm that detects densely connected regions (or clusters) of interaction networks. The MCODE works on vertex weighing by local neighborhood density (highest *k*-core) and outward traversal from a locally dense seed protein to separate the dense regions. A *k*-core is a graph of minimal degree *k* (graph G, for all *v* in G, deg(*v*) ≥ *k*). The highest *k*-core of a graph is the central, most densely connected subgraph (Bader and Hogue, 2003). Modules in a large network are functionally and statistically significant interacting clusters of nodes that resemble community organizations. Once the modules are found by MCODE, it becomes easy to find the hub genes (key regulator genes) in the network. These hubs genes are a part of the integrated network; they may be present in different and independent modules/sub-modules.

Such hub genes have many-fold roles; Firstly, they directly interact with the nodes in the module (in which they are present) to preserve the network’s stability and fast information processing with quick accessibility of the molecules. Secondly, the hub genes could be the most influencing nodes, becoming a strong cross-communication among different modules. It has been observed that each hub gene or specialized set of hub genes somehow controls a module that may constitute a functional process.

### Functional and Pathway Enrichment Analysis of Modules

We used the **g-Profiler** tool (Reimand et al., 2007) to perform comprehensive gene enrichment analysis or over-representation analysis (ORA) of our 86 target genes. It maps genes to known functional information sources and detects statistically significantly enriched terms. Besides, functional enrichment analysis of all modules were done by **Cluepedia** (Bindea et al., 2013) and **ClueGo** (Lee et al., 2005) tools to perform comprehensive Gene Ontology (GO)-enrichment analysis of each module. It integrates GO terms divided into three classes, namely biological process, molecular functions, and biological pathways among high centrality nodes (genes) as well as KEGG/BioCarta pathways and creates a functionally organized GO/pathway term network.

### Drug-Target Interactions

To determine the drug-target interactions, we integrated the DGIdb database (Freshour et al., 2021) (www.dgidb.org). The DGIdb is a web resource that provides information on drug-gene interactions and druggable genes from related publications and databases. We used 86 genes (key regulators) and their respective drugs from the DGIdb database. We built a drug-target bipartite network composed of drugs and target genes linked by experimentally validated drug-target binary associations. The network integration of these parameters makes it possible to infer whether two drugs share a common target. The list of target genes and interacting drugs is given in the Supporting Information (Supplementary Material S3).

## Result

### Disease-Associated Genes of TB and Its Overlapping NCDs

**FIGURE 2:**
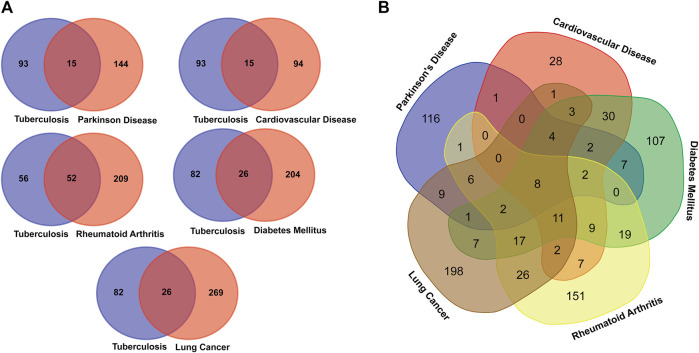
Venn diagram showing the number of overlapped genes among the TB and overlapping NCDs. **(A)** Association between Tuberculosis and NCDs. **(B)**. Overall disease gene association among the MTB and NCDs.

After comparing the disease-associated TB genes and Its overlapping NCDs presented in Table 1, we found that the 26 genes of DM, 52 genes of RA, 15 genes of CVD, 15 genes of PD, and 26 genes of LC overlapped with TB associated genes. Moreover, many disease genes are common among NCDs (Figure 2).

**TABLE 1:** List of disease-associated genes.

Tuberculosis	Parkinson Disease	Cardiovascular Disease	Diabetes mellitus	Rheumatoid arthritis	Lung cancer
IFNG, RNF34, SLC11A1, TNF, NCAPG2, RHOF, IL10, MT1JP, ESAT, TLR2, VDR, MBL2, HSPD1, IL1B, CCL2, SP110, NAT2, IL4, CFP, CAT, IL12B, CD14, HLA-DRB1, IRGM, IL2, IL6, CD209, TLR4, CXCL10, CXCL8, INHA, P2RX7, BCAR1, MMP1, CYP2B6, TSC1, TSC2, IFNGR1, MMP9, TLR1, CYP2E1, IL1A, IL17A, NOD2, CCL5, CTNND1, CSE1L, CSF2, TIRAP, ESX1, NOS2, IL17D, TGFB1, TLR9, ELF3, IL15, FOXP3, BMS1, IL12RB1, MAPK1, CORO1A, IRF1, IL22, CISH, SOCS3, IL23A, HSPA4, ACACA, MAPK14, TRBV20OR9-2, HP, HPD, WNT3, CHP1, INTS4, CD9, NLRP3, LAMC2, GC, GSTM1, GRAP2, VSX1, AGO2, HLA-C, HLA-B, RNF19A, IL18, STAT3, TMED2, GSTT1, HLA-A, IL27, TPPP, FCGR3B, FCGR3A, CRK, POLDIP2, CCR5, MYD88, AHSA1, DEFB4A, SOCS1, NRSN1, AIMP2, RBM45, WISP3 and CD27	LRRK2, SNCA, PINK1, PRKN, GBA, MAPT, PARK7, GDNF, SLC6A3, CYP2D6, UCHL1, APOE, BDNF, VPS35, TH, MAOB, ATP13A2, DRD2, NR4A2, COMT, PTEN, MUL1, CBLL2, SNCAIP, CHM, FYN, SYNM, BST1, HTRA2, PARK16, GSTM1, SOD1, NRTN, TNF, GCH1, EIF4G1, ABCB1, DDC, SLC18A2, MAOA, GNAL, GAK, PITX3, NTF3, GIGYF2, HMOX1, BAP1, LINGO1, NFE2L2, PON1, SNCB, HLA-DRA, GRN, ATXN2, GSK3B, NAT2, FMR1, FGF20, PARK10, HFE, C9orf72, CP, HSPA9, RIT2, APP, CSF2, CYP2B6, DRD3, LAMC2, MPZ, PARK3, MPHOSPH6, TARDBP, SLC41A1, FBXO7, NOS1, SOD2, PRKRA, HSPA8, IL1B, MCCC1, GAD1, POLG, DNM1L, PPARGC1A, HTT, CYP2E1, CCDC62, LINC02210-CRHR1, ADORA2A, ALDH1A1, GABPA, ATXN3, GSTP1, CHCHD2, RAB39B, DCTN1, HSPA4, STK39, TMEM175, UBE2K, LY6E, MTHFR, PRNP, TREM2, GFAP, PLA2G6, IL6, DNAJC6, SPR, FAM47E, FAM47E-STBD1, LINC02210, ANG, SCARB2, CTSD, DBH, ESR1, GPR37, HNMT, IL10, LMX1A, NOS2, SMPD1, VDR, RAB29, DNAJC13, USP24, HPGDS, UBE2S, LRRK1, DRD1, SLC30A10, TBP, TMEM230, LYST, TAF1, OPA3, LAMP3, STH, SIPA1L2, DGKQ, NSF, WNT3, KANSL1, ALDH2, CALB1, CASP3, CASP9, CDK5, CNR1, CYP1A2, ESR2, MAPK1, RET, VEGFA, MANF, SIRT2 and MIR133B	ACE, CRP, APOE, MTHFR, LPA, REN, NOS3, PON1, AGT, IL6, SERPINE1, CETP, TNF, LDLR, HP, LPL, APOB, ABCA1, EEF1A2, AGTR1, VEGFA, PCSK9, APOA1, TNFRSF11B, ICAM1, ALB, VWF, LEP, ADIPOQ, APOC3, IGF1, F7, FTO, PLG, PLA2G7, CCL2, PTGS2, PPARA, PPARG, NPPB, MPO, AGER, ADM, ESR1, APOA5, HMOX1, ACE2, VCAM1, CBS, GDF15, TLR4, RETN, IL18, MMP9, OR10A4, EDN1, CYP2C19, ALOX5, MBL2, VDR, F3, EPHX2, CST3, ADRB1, SELE, ANGPT2, TGFB1, IL10, ABCG8, OLR1, PLA2G2A, NFE2L2, ALDH2, PON2, GABPA, APOL1, MMP2, KNG1, IL1B, SELP, DECR1, FGB, ADRB2, PGR-AS1, PLA2G1B, CD14, HFE, F2, VPS51, RBP4, NPY, GPX1, BDNF, UTS2, NR3C1, COX2, HMGCR, NR3C2, GNB3, SIRT1, CYBA, TCF7L2, CDKN2A, MIR21, HLA-DRB1, FGF23, CCR5 and PLA2G6	INS, HNF4A, GCK, HNF1A, PPARG, ATN1, APOE, TCF7L2, KCNJ11, CRP, ACE, GAD2, ABCC8, GCG, HLA-DQB1, VEGFA, INSR, HLA-DRB1, ADIPOQ, IL6, LEP, PON1, PDX1, HNF1B, HP, AGER, ALB, TNF, SLC30A8, SERPINE1, GAD1, FTO, IGF1, WFS1, HLA-C, IRS1, UCP2, REN, AGT, CAPN10, SIRT1, PPARA, NOS3, GLP1R, SLC30A10, RBM45, OR10A4, FN1, IAPP, RENBP, CCL2, SLC2A4, TXNIP, PPARGC1A, HFE, LOC102723407, CAT, IRS2, RETN, AGTR1, AKR1B1, LPA, NEUROD1, VDR, LMNA, MMP9, NFE2L2, EHMT1, ZGLP1, CD36, CTLA4, EDN1, EEF1A2, HLA-A, IDE, IL4, CDKAL1, DECR1, IL18, LPL, MTHFR, ENPP1, SLC2A2, SREBF1, LEPR, TP53, GCKR, CETP, DPP4, HLA-DQA1, HMOX1, IFNG, PTPN1, MOK, RBP4, TRBV20OR9-2, UCP3, INSM2, ADRB3, APOA1, APRT, CD34, CTGF, GABPA, IL1A, IL1B, IL10, KCNQ1, MMP2, PTPRN, SLC5A2, TLR4, ADIPOR1, ADIPOR2, IL2RA, NR0B2, ABCA1, ALDH2, CDKN2A, GLUL, IGF2, TNFRSF11B, PIK3CA, PIK3CB, PIK3CD, PIK3CG, SOD2, UCP1, FGF21, G6PC2, PTGS2, AOC3, FXN, ACP1, APOB, BDNF, CEL, GIP, GPX1, LDLR, MTNR1B, PAX4, SHBG, ST3GAL4, SLC2A1, TGFB1, EIF2AK3, PTPN22, APOA5, CP, POMC, SOD1, PTEN, ATM, LIPC, ADA, ADM, CD59, DDIT3, DMPK, FABP2, GCGR, GLO1, HGF, HMGB1, HMGCR, IFNA1, IFNA13, IGFBP3, IL1RN, MC4R, NFKB1, PLG, PON2, MAPK8, SGK1, SPP1, TCF7, TXN, PDHX, KLF11, IGF2BP2, MIR146A, CYBB, ICA1, ABCG2, GATA6, SLC19A2, ADH1B, AHSG, AOC2, APP, CFTR, CST3, DPT, EGFR, EPO, ESR1, FOXO1, GGT1, GH1, GHR, GSTM1, HIF1A, HLA-B, IGF1R, IGFBP2, IL2, ISG20, KCNA3, MBL2, MPO, NGF, NOS2, PAX6, PCK1, PPIA, PRKAA1, PRKAA2, PRKAB1, VWF, APOL1, NAMPT, SOSTDC1, NEUROG3, FOXP3, GHRL, ACE2 and PPARGC1B	TNF, HLA-DRB1, IL6, PTPN22, IL1B, RBM45, IL10, PADI4, CRP, IL17A, CRYGD, CXCL8, IFNG, IL1A, STAT4, VEGFA, CTLA4, IL1RN, MTHFR, IL18, TRAF1, CD28, TNFAIP3, MMP1, TLR4, CSF2, IL2, TP53, IRF5, IL4, TNFSF11, NFKB1, PTGS2, CD40, HLA-DPB1, TNFRSF11B, IL2RA, TLR2, MMP3, FCGR3A, MBL2, FCGR2A, CCL2, TRBV20OR9-2, MMP2, FOXP3, STAT3, SLC22A4, IL23A, IL15, MMP9, TNFRSF1B, FCRL3, CD14, MAPK1, CCR6, IL6R, MIF, MMP13, VCAM1, VIM, MIR146A, CIITA, HLA-C, CCR5, FCGR3B, ICAM1, ABCB1, MAPK14, TGFB1, TNFRSF1A, IL32, NLRP3, MIR155, CXCR4, BCL2, CCL5, CXCL12, IL22, ENO1, SLC11A1, CD40LG, CRH, PRTN3, ISG20, CD68, FN1, HIF1A, SAA1, TNFSF13B, LOC105369230, STAT1, NR3C1, MMP14, CHI3L1, CRK, CXCL10, SPP1, AIMP2, GRAP2, AHSA1, RNF19A, POLDIP2, AGER, NFKBIL1, ACAN, CCL21, ZFP36, CD44, GPI, COX2, PIK3CD, PIK3CG, VDR, CDR3, IL21, REL, RUNX1, CYR61, TNFSF14, FAS, ESR1, PDCD1, PML, MAPK8, MIR223, IL6ST, AFF3, PTPRC, TRAF6, TNFRSF14, BLK, ACP5, HLA-DQB1, TAP2, NAT2, BSG, HLA-A, HMGB1, SERPINA1, PIK3CA, PIK3CB, CCL20, SELE, TNFSF15, HPGDS, IL33, LOC102723407, AHR, HLA-DMB, C5orf30, C6orf10, AR, MS4A1, FGF2, FOS, CXCR3, GSTM1, IL4R, IL7, JUN, NM, RARA, TLR3, TYMS, VIP, TNFRSF11A, PADI2, CARD8, IL17C, MBL3P, IL17D, KRT20, HT, WG, PTPN2, TYK2, MMEL1, ALOX5, CAT, CDK6, ADIPOQ, TAGAP, NCF1, PRKCQ, SOD2, C5, MICA, ACP1, PARP1, CDH11, CSF1, EPHB2, F2RL1, HLA-DRB4, IGF1, IL13, LTA, MEFV, MTX1, OSM, PLA2G1B, SLC19A1, THBS1, TIMP1, PTGES, DKK1, ICOS, NOD2, AGBL2, PRAM1, IL2RB, ANKRD55, SPRED2, FASLG, CTGF, DHFR, IRAK1, PON1, PRDM1, MPO, ZAP70, HLA-DQA1, NOTCH4, PHF19, BTNL2, ANGPT1, APOE, BTF3P11, CASP3, CD34, CDKN1A, CDKN2A, CREB1, EGFR, FCGR2B, FOXO3, FLT1, CFH, HLA-DMA, IFNA13, IGF2, IL16, KDR, LPA, NR4A2, PLG, SAA@, TAC1, ADAM17, TRB, NR1I2, SOCS3, CLOCK, LRPPRC, CXCL13, SIRT1, RETN, IL17F, SLCO6A1 and GSTK1	EGFR, TP53, KRAS, ALK, GSTM1, CYP1A1, CDKN2A, ERBB2, PTGS2, VEGFA, MET, XRCC1, GSTT1, TERT, EGF, FHIT, CHRNA3, BCL2, STAT3, CHRNA5, HPGDS, ERCC2, OGG1, ABCB1, TNF, PIK3CA, AKT1, BRAF, CCND1, GSTP1, RASSF1, IL6, STK11, NFE2L2, TSC1, SLCO6A1, GSTK1, CLPTM1L, MMP9, NFKB1, CYP2E1, MPO, PTEN, EPHX1, HGF, MMP2, CYP2A6, HIF1A, MYC, TGFB1, CHRNB4, GABPA, IGF1R, MDM2, COX2, PPARG, EML4, ERCC1, NQO1, TNFSF10, PIK3CB, CYP2B6, CYP2D6, PIK3CD, PIK3CG, RET, ROS1, XRCC3, IL24, COPD, APEX1, ATM, MGMT, MIR21, GSTM2, MMP1, ABCC1, PTHLH, ABCG2, CAV1, CXCL8, PCNA, NKX2-1, NAT2, CD44, FGFR1, IL1B, MUC1, TP73, XPC, PROM1, KEAP1, APC, ASCL1, CASP3, FN1, HRAS, IGF1, MCL1, MTHFR, SOD2, CD274, ARHGAP24, TP63, CDH1, CEACAM5, CHRNA4, EZH2, EPCAM, RARB, SPP1, TNFRSF10B, TBC1D9, MALAT1, HYKK, CDK2, CDKN1A, CYP1B1, ERBB3, ESR1, HNRNPA2B1, MAP2K7, CCL2, VIM, CHEK2, FAS, CRYZ, CTNND1, MTOR, FUS, IFNG, MAPK1, SOX2, TWIST1, TYMS, CXCR4, MIR155, AHR, BIRC5, CSF2, CYP1A2, DNMT3B, EGR1, HSP90AA1, ITK, MAPK8, SEMA3F, SLC22A3, SMARCA4, SP1, ZEB1, TUSC2, WWOX, PARP1, CTNNB1, DNMT1, ESR2, GLB1, GPX1, IGFBP3, IL10, MLH1, NME1, PXN, VEGFC, XPA, ABCC3, EPB41L3, SIRT1, CADM1, SEMA6A, UCN3, BRCA2, IREB2, BSG, CALCA, DMBT1, EPHB2, FOXM1, FOXO3, GAPDH, GRP, HMOX1, ICAM1, IL2, IL17A, KRT19, MSH3, SERPINE1, SERPINA1, PML, PR@, MAPK3, MAP2K1, RAD51, CXCL12, HDAC9, BCL2L11, PPP1R13L, GADD45G, SLC12A9, MARCKSL1, WLS, MIR31, MIR34A, AXL, BAX, CDK4, CTGF, CYP24A1, ELANE, HSPA4, SMAD4, MCC, MDM4, MMP7, MSH2, MYCL, NBN, NOTCH1, PRDX1, RAC1, RRM1, S100A2, SHOX2, SKP2, AURKA, TGFBR2, VDR, SCLC1, TFPI2, ADAM9, YAP1, TDGF1P6, SESN2, MIR146A, MIR182, MIR205, MIR210, NOTCH3, FEN1, ACTN4, AGER, BDNF, BRCA1, CASP9, CAT, CDH13, CDKN1B, CHEK1, CHRNA1, COL11A2, CLDN7, CRP, AKR1C1, DVL3, EPHA2, ENO2, ERBB4, ERCC5, FGF2, FUT4, HDAC1, NRG1, HSPB1, TNC, IFNB1, IGF2, IGFBP2, CD82, SMAD2, SMAD3, MEN1, MMP12, MMP13, MST1R, MTAP, MUC4, PAH, PKM, PLK1, PRRX1, POU5F1, PTN, ROBO1, SLC2A1, SOX4, TGM2, TIMP1, TXNRD1, AIMP2, SETD2, PRR11, AKR1B10 and CTCFL

### Construction of Network and Characterization of Topological Properties

**FIGURE 3:**
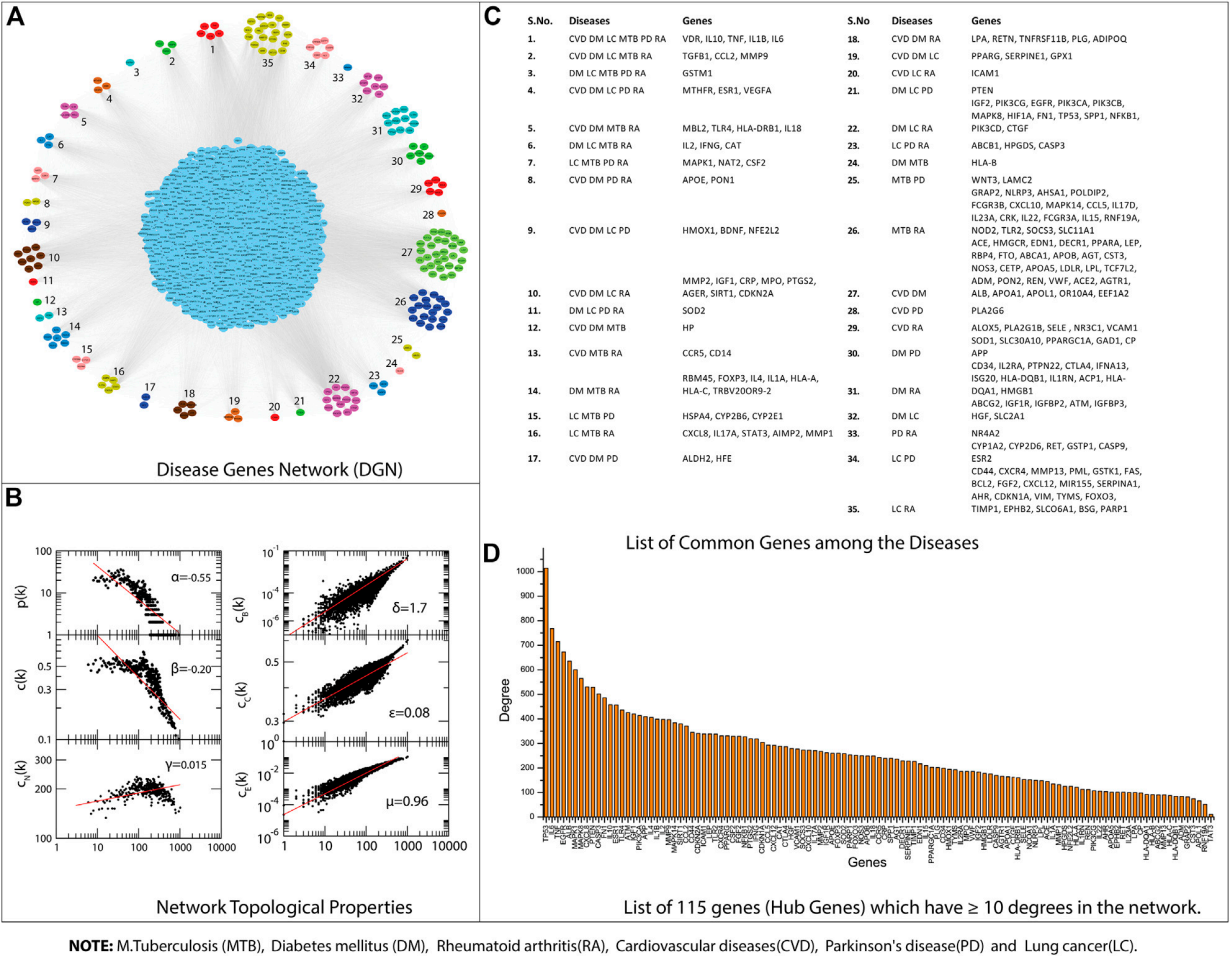
**(A)** Disease Gene Network (DGN). **(B)** DGN topological properties. **(C)** List of common genes among the Diseases, e.g., tuberculosis (TB), diabetes mellitus (DM), rheumatoid arthritis (RA), cardiovascular diseases (CVD), Parkinson’s disease (PD), and lung cancer (LC). **(D)** List of 115 genes that have ≥10° degrees in the network.

All the disease-associated genes of TB, PD, CVD, DM, RA, and LC were used to construct their gene interaction networks. We constructed six networks for each disease and then merged them into a single network, i.e., disease-gene (DG) network. Next, we measured the topological properties of the network. The probability of degree distributions *P(k), average* clustering coefficient *C(k),* and neighborhood connectivity *CN(k)* showed the fractal nature of the network, which is a self-organization property of the network where the network maintains the nature of nodes at various levels and not follow the centrality-lethality control system (removing of one or more hubs does not cause network breakdown) (Nafis et al., 2016). The network behavior indicated a hierarchical scale-free network, and all the topological properties of networks followed the power-law distributions (Pastor-Satorras et al., 2001; Ravasz and Barabási, 2003). The power-law fitting on the topological properties data points was performed using the standard statistical fitting method given by Clauset et al.(Clauset et al., 2009). The negative values of *P(k)* and *C(k)* indicated that the network followed the hierarchical pattern, while the positive value of *CN(k)* implied that the network has the assortativity that recognizes the clusters (rich clubs) regulating the network. The network centrality measurements (C_B_(k) and C_C_(k)) show the information flow in the network and anticipate the most influential nodes. Next, the C_E_(k) characterized the well connectedness of nodes in the network and calculated the efficacy of the unfurl data of nodes from the network. Besides network centrality, we also measured the node centrality in the network. A node with the higher centralities value can help recognize a biological entity (genes) with the most important role in the network (Jeong et al., 2001; Hahn and Kern, 2005). We selected nodes with at least ≥10° because previously reported that degree centrality (specifically for undirected networks) is an effective measure since many nodes with high degrees also have high centrality by other measures. The higher the degree, the more central the node is (Jeong et al., 2001; Wagner and Fell, 2001; Ma and Zeng, 2003; Bergmann et al., 2004; Hahn and Kern, 2005; Golbeck, 2013, 2015; Ashtiani et al., 2018); like this, we have identified 115 genes with higher degrees in the network. The complete details of the DG-network and its topological properties, gene association among the diseases, and hub genes DG-network are given in Figure 3.

### Modules/Clusters in Gene Interaction Network

**FIGURE 4:**
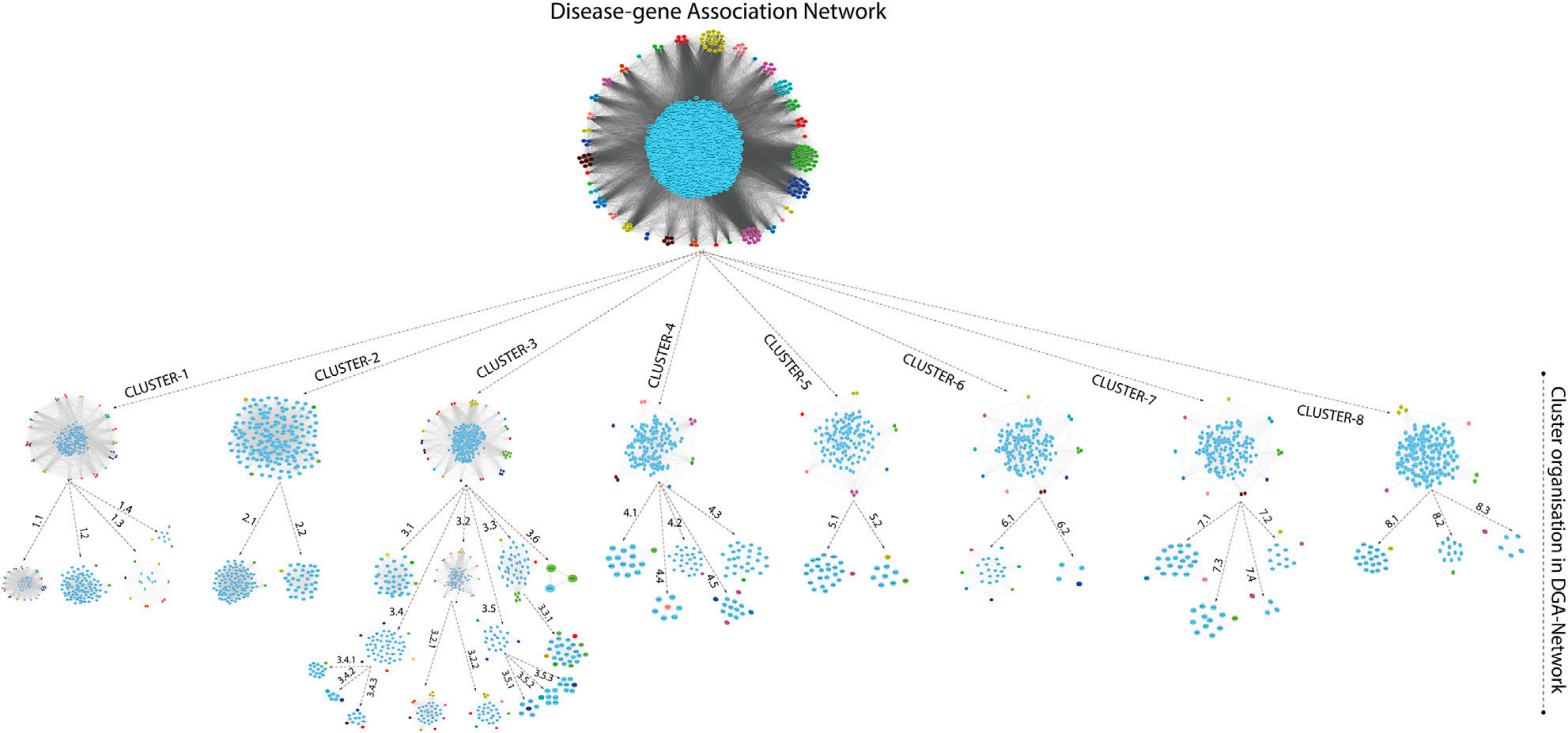
Modules and sub-modules of the main network (Disease-genes network).

**FIGURE 5:**
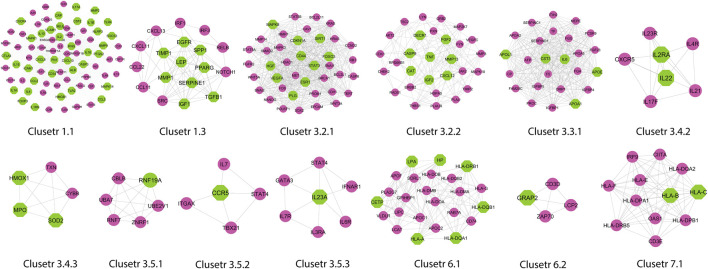
Important modules (including motifs and rich clubs) in the network. These functional modules are common in tuberculosis and its associated NCDs.

A novel graph-theoretic clustering algorithm, MCODE, detects the densely connected regions (or clusters) of interaction networks called a module. Modules in a large network are functionally and statistically significant interacting clusters of nodes that resemble community organizations in the network. In our study, eight modules (high scoring) were subjected from the MCODE, which further descended to sub-modules to reach up to hub genes (Figure 4). Next, we start gene tracing to access the regulation of the network; the gene tracing was done purely on the appearance of the target genes (genes with ≥10° in the networks) in various sub-modules. Importantly, we selected only those sub-modules which contain our target genes, and the rest of the sub-modules were eliminated from the study. As the results, we identified a total of 33 high-scoring significant modules, but more specifically, we considered only 13 modules (Figure 5) that contained only 86 hub genes, which are common in TB and overlapping NCDs; The details are given in Table 2.

**TABLE 2:** List of 13 Modules and Sub-Modules which contain hub genes of Disease (MTB, DM, CVD, LC, RA, and PD) but we only considered those modules (✔) which have hub genes that are common in MTB as well as overlapping NCDs.

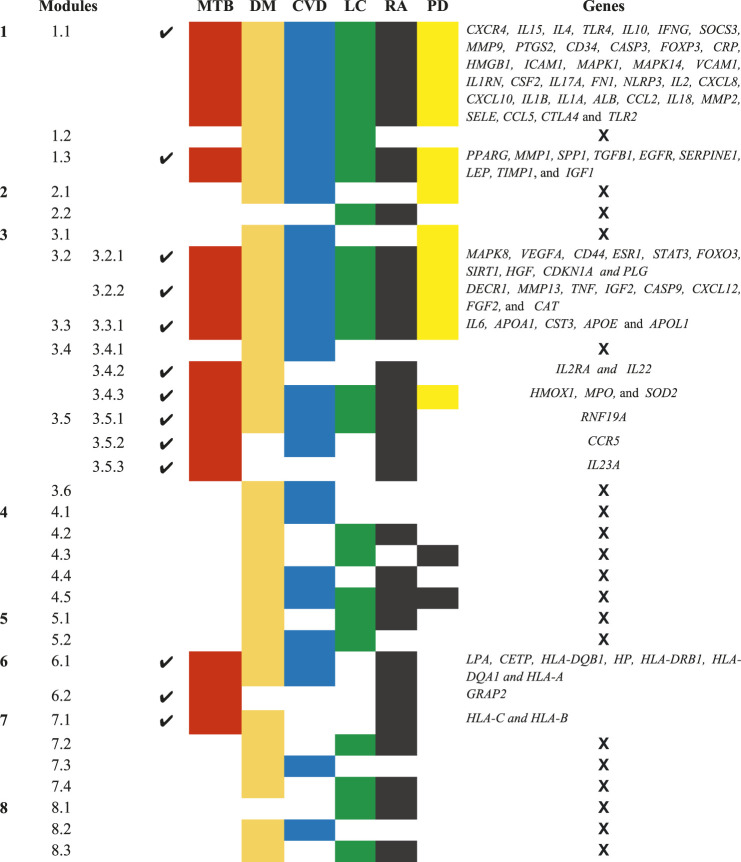

Besides, other 20 significant modules that contain several hub genes, which are also deeply rooted in the network and have the ability to reach from the main network to the rich club (hub genes) through various levels of the organizations via modules and sub-modules and work at the grassroots level with basic maintaining technologies and are generally the backbone of keeping network stability. In the future, these hub genes can be used to find the disease-gene relationship, find the multiple side effects of unrelated drugs using drug-target network, drug-repurposing opportunities, and can investigate new targets and multidrug treatment among the NCDs (*such as* Parkinson’s disease, cardiovascular disease, diabetes mellitus, rheumatoid arthritis, and lung cancer). All these 33 significant modules are given in Supplementary Material S1.

### GO Enrichment Analysis

**FIGURE 6:**
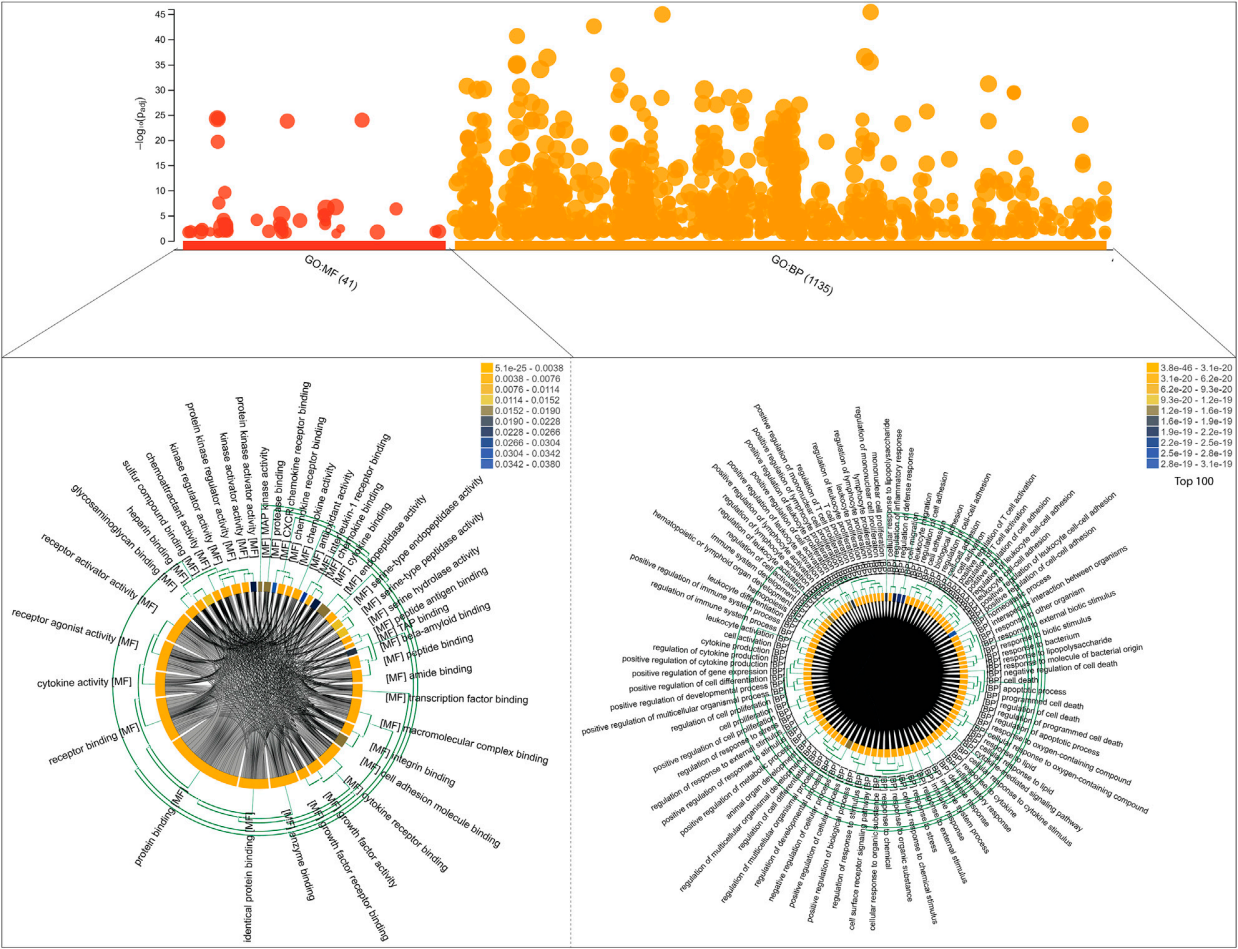
Functional enrichment analysis of 86 target genes, including molecular functions and biological processes, is shown on the bubble graph based on log10(P_adj_) values in the *Y* axis. The clustering of Gene Ontology (molecular functions and biological processes) is shown in chord diagrams for target genes.

We identified a total number of 86 key regulators that were commonly associated with TB and other NCDs that are critically involved many biological processes including cellular response to cytokine stimulus, response to cytokine, cytokine-mediated signaling pathway, inflammatory response, cellular response to chemical stimulus, response to organic substance, response to stress, Défense response, response to external stimulus, regulation of cell-cell adhesion, cell activation, cell surface receptor signaling pathway, regulation of immune system process, immune system process and regulation of cell adhesion. These target genes are also enriched with a certain molecular function that are cytokine activity, signaling receptor binding, receptor ligand activity, cytokine receptor binding, growth factor activity, integrin binding, enzyme binding, chemoattractant activity, peptide binding, glycosaminoglycan binding, antioxidant activity, kinase regulator activity, chemokine receptor binding and transcription factor binding. The g:Profiler analysis is shown in Figure 6.

**FIGURE 7:**
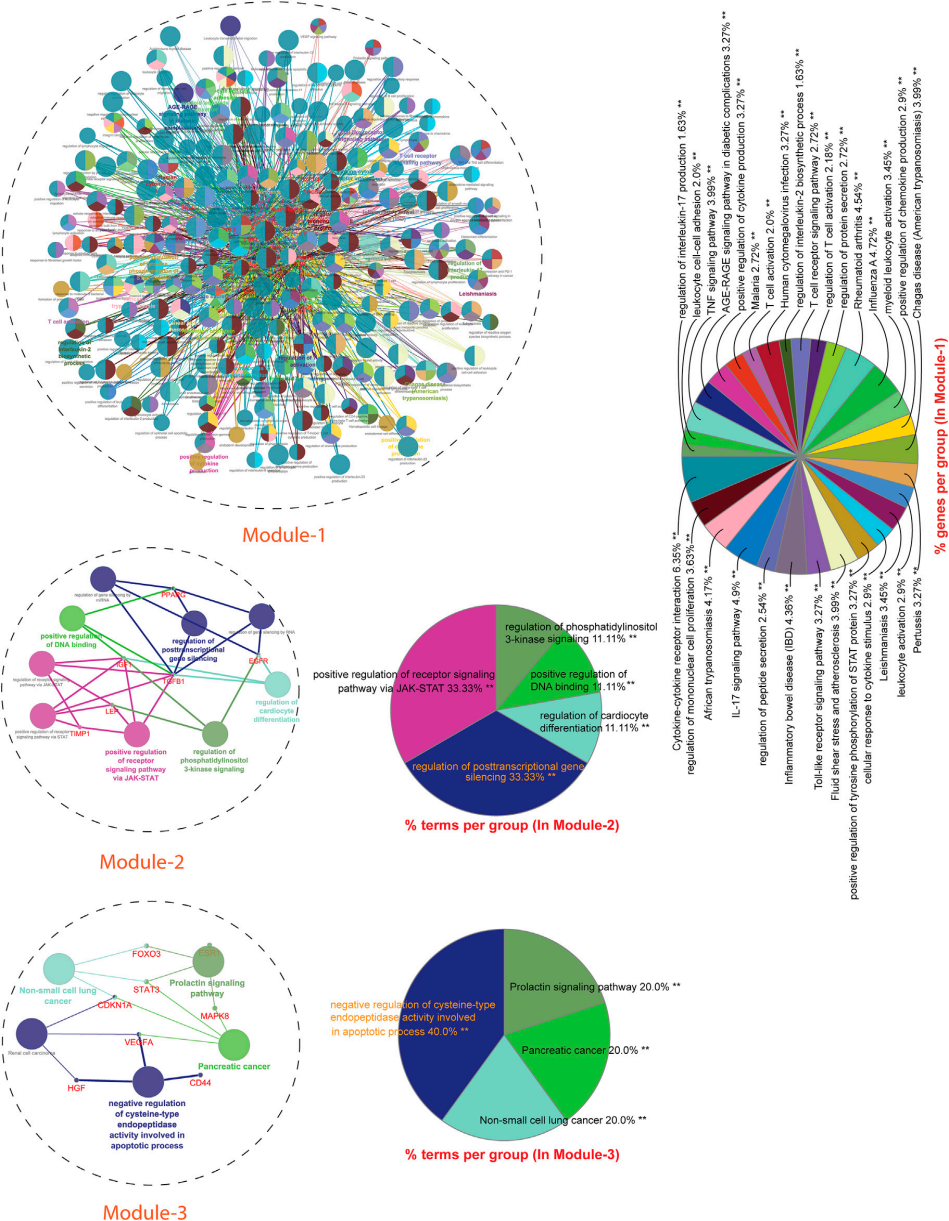
GO enrichment Analysis: Network representation shows the various biological processes and pathways enriched by genes of module-1 to module-3. Each node represents a pathway and biological process. The node size reflects the enrichment significance of pathway and biological processes. Node color shows the class that they belong to. Mixed coloring means that the particular node belongs to multiple classes.

**FIGURE 8:**
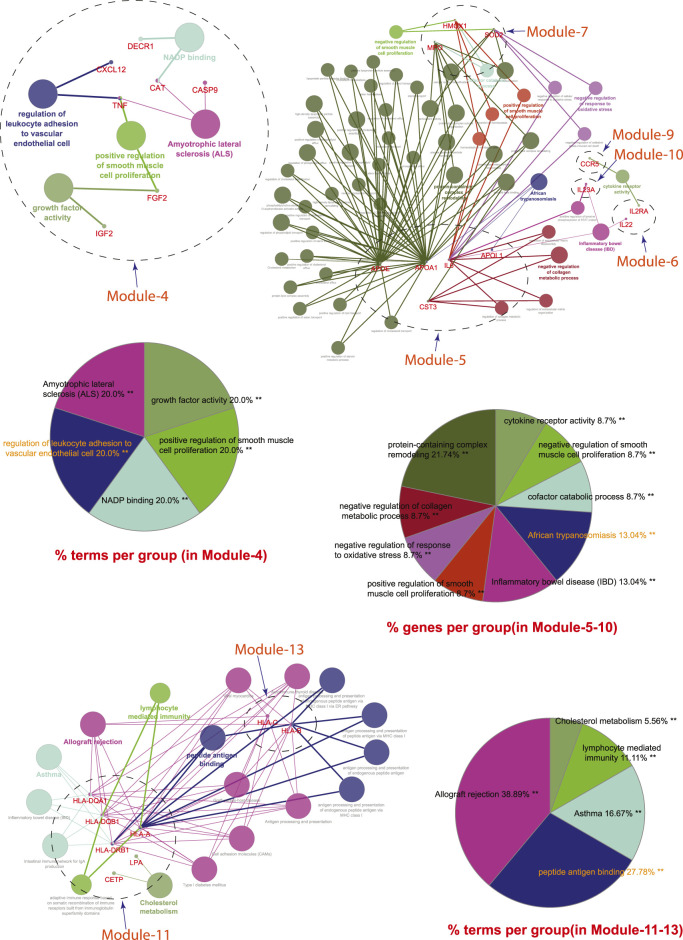
GO enrichment Analysis: Network representation shows the various biological processes and pathways enriched by genes of module-4 to module-13. Each node represents a pathway and biological process. The node size reflects the enrichment significance of pathway and biological processes. Node color shows the class that they belong to. Mixed coloring means that the particular node belongs to multiple classes.

Next, we performed a comprehensive functional analysis of 13 modules using the ClueGO and Cluepedia tool (Cytoscape plugin), which integrates Gene Ontology (GO) terms and KEGG/BioCarta pathways. The GO analysis of each modules reflects their involvement in many important biological processes and in diverse biological pathways, details are given in Figures 7, 8.

Module-1 is the largest and highly-interconnected module among 13 modules, containing 36 key regulators (*CXCR4*, *IL15*, *IL4*, *TLR4*, *IL10*, *IFNG*, *SOCS3*, *MMP9*, *PTGS2*, *CD34*, *CASP3*, *FOXP3*, *CRP*, *HMGB1*, *ICAM1*, *MAPK1*, *MAPK14*, *VCAM1*, *IL1RN*, *CSF2*, *IL17A*, *FN1*, *NLRP3*, *IL2*, *CXCL8*, *CXCL10*, *IL1B*, *IL1A*, *ALB*, *CCL2*, *IL18*, *MMP2*, *SELE*, *CCL5*, *CTLA4*, and *TLR2*) associated with TB and other five overlapping NCDs (PD, CVD, DM, RA, and LC). We observed that module-1 is statistically enriched by diverse biological processes and pathways, including cytokine-cytokine receptor interaction, T-cell activation, leukocyte activation, IL17, and TNF signaling pathways, Toll-like receptor signaling pathways, cellular responses to cytokine stimulus, rheumatoid arthritis, AGE-RAGE signaling pathways in diabetic complication and positive regulation of cytokine production.
Module-2 contains nine key regulators (*PPARG, MMP1, SPP1, TGFβ1, EGFR, SERPINE1, LEP, TIMP1,* and *IGF1*) associated with TB and other five overlapping NCDs were enriched by biological processes and pathways, namely regulation of gene silencing by miRNA, regulation of gene silencing by RNA, regulation of post-transcriptional gene silencing, positive regulation of DNA binding, regulation of receptor signaling pathway by JAK-STAT, positive regulation of receptor signaling pathway by STAT, positive regulation of receptor signaling pathway by JAK-STAT, and regulation of cardiocyte differentiation.
Module-3 contains ten key regulators (*MAPK8, VEGFA, CD44, ESR1, STAT3, FOXO3, SIRT1, HGF, CDKN1A,* and *PLG*) associated with TB and other five overlapping NCDs were enriched by biological processes and pathways, namely prolactin signaling pathway, non-small cell lung cancer, renal cell carcinoma, pancreatic cancer, and negative regulation of cysteine-type endopeptidase activity involved in the apoptotic process.
Module-4 contains eight key regulators (*DECR1, MMP13, TNF, IGF2, CASP9, CXCL12, FGF2,* and *CAT*) associated with TB, and other five overlapping NCDs were enriched by biological processes and pathways, namely NADP binding, regulation of leukocyte adhesion to the vascular endothelial cell, amyotrophic lateral sclerosis (ALS), positive regulation of smooth muscle proliferation, and growth factor activity.
Module-5 contains five key regulators (*IL6, APOA1, CST3, APOE,* and *APOL1*) associated with TB, and other five overlapping NCDs were enriched by diverse biological processes and pathways, including negative regulation of collagen metabolic process, protein-containing complex remodeling, African trypanosomiasis and inflammatory bowel disease.
Module-6 containing two key regulators (*IL2RA* and *IL22*) associated with TB and the other two overlapping NCDs (DM and RA) were enriched by biological processes and pathways, namely inflammatory bowel disease and cytokine receptor activity.
Module-7 contains three key regulators (*HMOX1*, *MPO*, and *SOD2*) associated with TB, and other five overlapping NCDs were enriched by biological processes and pathways, namely negative regulation of smooth muscle proliferation, negative regulation of response to oxidative stress, positive regulation of smooth muscle proliferation and cofactor catabolic process.
Module-8 containing one key regulator (*RFN-19A*) associated with TB and the other four overlapping NCDs (CVD, DM, RA, and LC) did not show any enrichment in biological processes and pathways.
Module-9 containing one key regulator (*CCR5*) associated with TB and the other two overlapping NCDs (CVD and RA) was enriched by biological processes and pathways, namely cytokine receptor activity.
Module-10 containing one key regulator (*IL23A*) associated with TB and another overlapping NCD (RA), was enriched by biological processes and pathways, namely regulation of phosphorylation of STAT protein and inflammatory bowel disease.
Module-11 containing seven key regulators (*LPA*, *CETP*, *HLA-DQB1*, *HP*, *HLA-DRB1*, *HLA-DQA1*, and *HLA-A*) associated with TB and other three overlapping NCDs (DM, CVD, and RA) were enriched by biological processes and pathways, namely cholesterol metabolism, type-1 diabetes mellitus, asthma, allograft rejection, peptide antigen binding, graft-versus-host disease, inflammatory bowel disease, cell adhesion molecules, the intestinal immune network for IgA production, and adaptive immune response based on somatic recombination of immune receptors built from immunoglobin superfamily receptors.
Module-12 containing one key regulator (*GRAP2*) associated with TB and another one NCD (RA), did not show any enrichment in biological processes and pathways.
Module-13 containing two key regulators (*HLA-C* and *HLA-B*) associated with TB and two overlapping NCDs (DM and RA) were enriched by biological processes and pathways, namely viral myocarditis, autoimmune thyroid disease, peptide antigen binding, graft-versus-host disease, and antigen processing and presentation.

### Drug-Target Interactions

**FIGURE 9:**
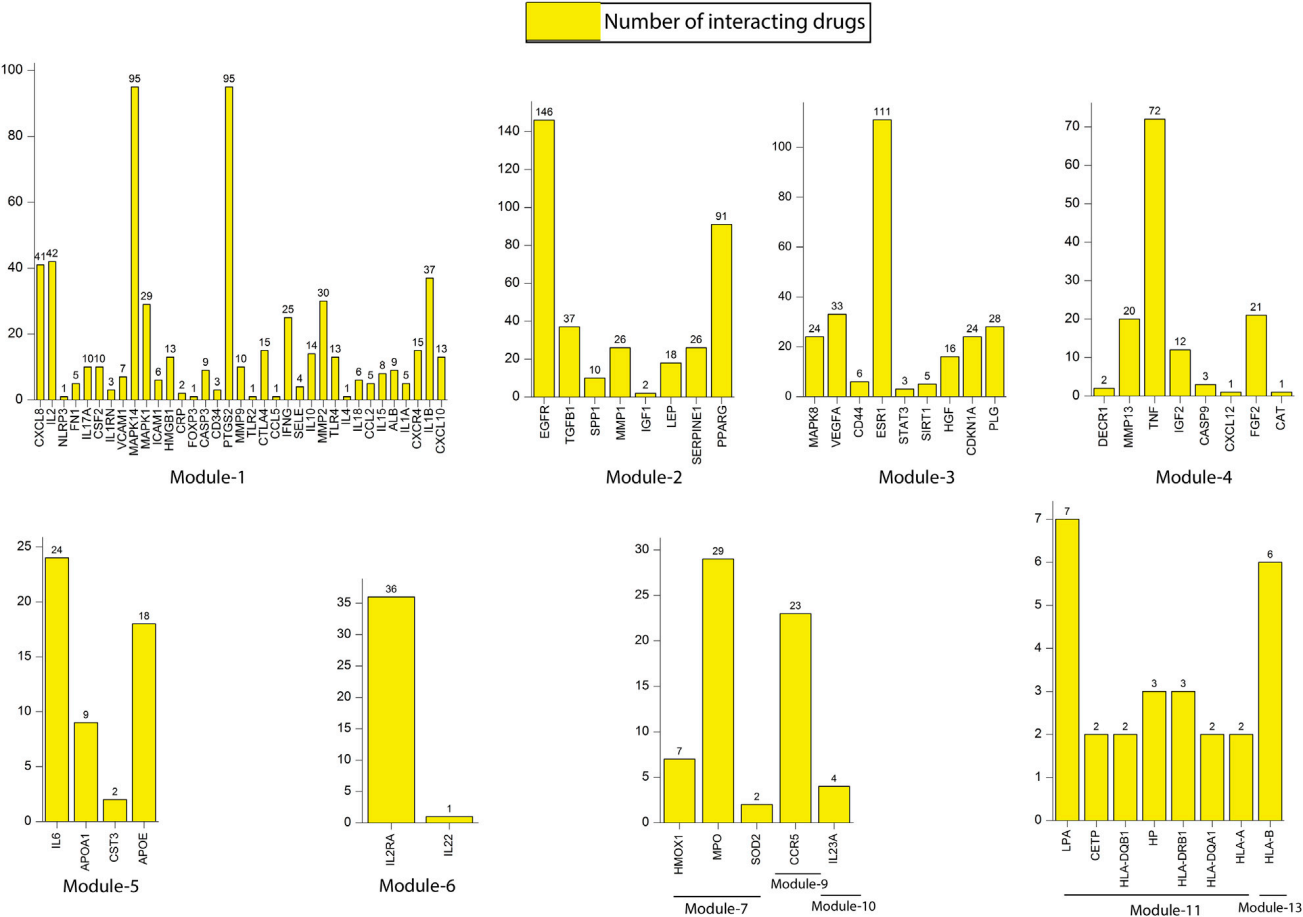
Representation of the number of interacting drugs with key regulators.

We constructed a bipartite network (two classes of nodes: drugs and genes) by mapping all the 86 key regulators from 13 modules to their respective drugs from the DGIdb database **(** Supplementary Table S1 **)**. Results revealed that all most all the key regulators found their hits except few genes, namely SOCS3 (module 1), TIMP1 (module 2), FOXO3 (module 3), APOL1 (module 5), *RNF19A* (module 8), *GRAP2* (module 12) and *HLA-C* (module 13). The number of interacting drugs with individual target genes (key regulators) is represented in Figure 9. A detailed list of target genes and their respective drugs are given in Supplementary Table S2. Results reflect that gene that interact with a larger number of drugs may be more related to the underlying mechanisms driving the pathological phenotype associated with these drugs. A fundamental network-based metric that can be used to identify such genes is their degree. The genes with a higher degree (interacting with drugs) are more likely to be related to tuberculosis and its associated NCDs.

## Discussion

The epidemiological shift in diseases, such as the occurrence of infectious diseases overlapping with other non-communicable diseases, is a critical health-related problem globally. One such convergence, included in the current study, is the occurrence of TB with its overlapping NCDs such as PD, CVD, DM, RA, and LC. It has been reported that TB patients have a 1.38-fold higher risk of developing PD as compared to control subjects. It is well documented that TB and CVD augment the risk of each other. DM is an important risk factor for TB and is associated with a 3-fold higher risk of contracting TB (Boutayeb, 2006; Leung et al., 2007). TB also has a bidirectional epidemiological association with RA and has reported that patients with RA have a 4-fold higher risk of developing TB than the control population (Carmona et al., 2003). The overlapping of TB with LC has been reported to increase lung cancer risk and vice versa. This epidemiological shift involving overlapping different diseases in a common individual raises pharmacological issues and challenges related to their clinical co-management in the affected population. These issues emphasize finding common regulators (key regulators) of overlapping diseases that can be targeted commonly for their therapeutic management, irrespective of vertical approaches focused on individual diseases.

Keeping in mind the concept of a network-based approach, we emphasized building a disease network, reflecting genes commonly associated with TB and with one or more selected overlapping NCDs. Bipartite networking aimed to determine drug-target interactions revealed that all the 86 key genes found their hits except few genes namely *SOCS3, TIMP1, FOX O 3, APOL1, RNF19A, GRAP2*, and *HLA-C.* These findings provide us with insight into the overall molecular picture of these overlapping diseases and consider the fact that TB and these NCDs co-exist with each other at the gene level. Moreover, the findings also provide an insight to think of re-devising present strategies by looking at the collective effect of drugs on the genes commonly co-existing with TB and other overlapping NCDs.

We found that these 86 key regulators are enriched by diverse important biological processes and pathways, possibly connecting TB with these overlapping NCDs (Figures 6, 7). Scientific evidence originated from many studies that have justified the biological significance of the majority of these key genes/regulators. The CXCR4 surface expression has been found associated with TB. Macrophages in *in vitro* TB infection showed increased CXCR4 surface expression, whereas, in *in vivo*, amelioration of disease was associated with the reduction of CXCR4 expression of macrophages. Changes in CXCR4 expression in macrophages were found to occur due to the innate immune response against TB (Hoshino et al., 2004). Higher levels of *IL-15* have been reported in pulmonary TB and diabetes mellitus, reflecting their inflammatory characteristics. The level of IL-15 was also found significantly higher in RA (Muro et al., 2001) and CVD patients (Chang et al., 2006). The IL-4 has been shown correlated with TB susceptibility as well as with its progression (Lugo-Villarino et al., 2018). Binisor et al. reported increased levels of IL-4 in diabetic and non-diabetic obese individuals compared to healthy controls (Binisor and Moldovan, 2016). IL-4 levels are also associated with the development of early-stage of rheumatoid arthritis (Haikal et al., 2019). IL-4 producing Th2 cells has been more resistant to hypercholesterolemia, leading to atherosclerotic plaque stability (Frostegård et al., 1992). A higher level of *IL-4* has been reported in juvenile parkinsonism individuals as compared to controls (Mogi et al., 1996).

Moreover, IL-4 polymorphisms have been found linked with the risk of lung cancer (Tan et al., 2019). TLR4 is considered an important innate and adaptive immune response molecule against TB. This molecule has the ability to recognize *Mycobacterium tuberculosis* (*pattern recognition receptors (PRRs)*) and generate innate immune responses (Chang et al., 2006). Activation of TLR4 has been found to promote insulin resistance in DM and participate in its complications such as diabetic nephropathy, diabetic retinopathy, and diabetic vascular disease. TLR4 has been found involved in the deposition and scavenging of amyloid-beta and regulation of neuroinflammation in Alzheimer’s disease (Heneka et al., 2015). Elevated levels of *TLR4* along with *TLR2, TLR3*, and *TLR7* have been reported in RA synovium and in the dendritic cells of synovial fluid (Martin et al., 2003). In the brain, inflammation mediated by TLR4 is among the key factors responsible for PD-associated neurodegeneration (Amor et al., 2010).

Moreover, TLR4 activation has been reported to enhance many cytokines’ production and promote TRAF6 ubiquitination that facilitates LC cell migration and invasion (Zheng et al., 2020). IL-10 is an important molecule contributing to anti-mycobacterial host immunity and promotes *Mycobacterium tuberculosis* survival (Abdalla et al., 2016). Increased levels of IL-10 have been observed after stimulation by Ag85A *Mycobacterium. Tuberculosis* (Meenakshi et al., 2016). A higher level of IL-10 has been reported in the synovial fluid of RA patients, mediating neutrophil autophagy through the interaction of cytokine-cytokine receptors (An et al., 2018). One recent follow-up study revealed increased IL-10 expression associated with a higher risk of cardiovascular events (Rentzos et al., 2009; Yilmaz et al., 2014), reported that IL-10 is associated with PD’s pathogenetic mechanisms. Moreover, induced expression of IL-10R has been determined in metabolically restricted human lung adenocarcinoma cell lines, where it affects programmed death-1 protein leading to inhibition of tumour cell apoptosis.

It is known that IFN-γ is required to control the infection caused by *Mycobacterium Tuberculosis*. IFN-γ is secreted primarily by CD4^+^ T-cells as an adaptive response to infection (Knight et al., 2018). The level of these molecules has been found altered in the TB/diabetes mouse model (Meenakshi et al., 2016). A higher level of IFN-γ has been reported in coronary artery disease patients in comparison to healthy controls (Wang H. et al., 2019). The absence of IFN-γ has been found associated with the reduction of many PD-like features in IFN-γ deficient mice (Liscovitch and French, 2014). It has been reported that IFN-γ-mediated inhibition of lung cancer is regulated by PI3K-AKT signaling, correlating with PD-L1 expression (Gao et al., 2018). It has been observed that *Mycobacterium tuberculosis* infection induces the expression of SOCS3 in phagocytes, which in turn stops STAT3 activation by inhibiting some of the STAT3-activating cytokine receptors (Rottenberg and Carow, 2014), as well as proliferation and survival of lung adenocarcinoma cells (Speth et al., 2019). Increased SOCS3 expression has been found associated with RA (Meng et al., 2020), coronary artery disease (Zheng et al., 2020), and PD (Ng et al., 2019).

It has been reported that *Mycobacterium tuberculosis* inhibits caspase-3 leading to a reduction in macrophages apoptosis (Ali et al., 2020). Increased level of CD4+CD25+FOXP3+ T regulatory cells has been reported in Crohn’s disease and intestinal tuberculosis patients. Findings reflect that level of CD4+CD25+FOXP3+ T regulatory cells can be used as accurate biomarkers to differentiate both diseases. A higher level of C-Reactive protein has been reported in tuberculous lymphadenitis individuals (Kathamuthu et al., 2020), and its polymorphism has been found associated with a greater risk of developing PD (Wang et al., 2016). Altered regulation of nuclear protein HMGB1 has been found in Parkin expressing cells in PD and non-small cell lung cancer cells (Ayimugu et al., 2020; Qiu et al., 2020). IL17A has been found associated with the induction of autophagy in tuberculosis patients through a mechanism that activates MAPK1/3/14 (Tateosian et al., 2017). Elevated VCAM1 levels have been reported in patients with lung cancer, RA, and PD compared to their age-matched healthy controls (Navarro-Hernández et al., 2009; Tas et al., 2014; Perner et al., 2019). Activation of NLRP3 inflammasome has been reported associated with the pathogenesis of RA, cardiovascular diseases, and lung adenocarcinoma (Wang et al., 2016; Guo et al., 2018; Liu et al., 2018). TLR2, TLR4, and the NLRP3 inflammasome are also involved in inflammatory responses (Wada and Makino, 2016). The levels of CXCL8 were found elevated in pulmonary TB patients (Alessandri et al., 2006). Significant increases in MIG/CXCL6 and IP-10/CXCL10 have been suggested as a causative agent of diabetes in mammals (Capua et al., 2013). TNF-alpha, IL-1beta, CXCL8, and CXCL10 levels have been linked with ankylosing spondylitis and crystal, psoriatic and rheumatoid arthritis (Proost et al., 2006). PPARγ is a nuclear transcription factor activated by diverse endogenous and exogenous ligands, leading to cellular metabolism, proliferation, differentiation, and inflammation. Increased PPARγ expression has been reported in activated alveolar macrophages (AMs), a primary host cell in *Mycobacterium tuberculosis* infection. Impaired activity of PPARγ has been found in the setting of diabetes with and without cardiovascular diseases, PD, and LC cells (Mirza et al., 2015; Kwon et al., 2017; Bendaya et al., 2018; Lecca et al., 2018; Sippel et al., 2019)*.* Higher levels of TGF-beta1 have been reported in positive than negative tuberculin reactors in tuberculosis patients (Jang et al., 2006). TGFβ1/integrin β3 axis has been proposed as an anticipating target for combination therapy in EGFR-mutant lung cancer (Wang C. et al., 2019). Stoyney et al. found increased expression of *FABP3, FAS, FN1, IL1R2, LPL, SERPINE1, TGFB1*, and *VCAM1* and decreased expression of *SELPLG* and SERPINEB2 associated with hypertension and suggested that up-regulation of FAS, FN1, SERPINE1, TGFB1, and VCAM1 might be a reason for increased risk of cardiovascular diseases (Stoynev et al., 2014). Type 2 diabetes is associated with increased APOE, BAX, MMP1, NFKB1, PDGFB, SPP1, and TGFB2. CD44 acts as a macrophage binding site for the attachment of *Mycobacterium tuberculosis,* leading to macrophage recruitment against tuberculosis. Macrophage recruitment was found impaired in CD44-deficient (CD 44 (−/−)) mice. The role of CD74/CD44 MIF, a two-component receptor, has been determined in RA. The findings of this study suggested that its inhibition may offer a specific means to interfere with progressive joint destruction. Higher expression of SIRT1 and FOXO3 has been reported in diabetic patients and in rheumatoid arthritis synovial fibroblasts (Kok et al., 2013; Liu et al., 2015). The inhibition of AKT by shikonin activated the forkhead box (FOX)O3a/early growth response protein (EGR)1 signaling cascade and enhanced the expression of the target gene Bim, leading to apoptosis in lung cancer cells (Jeung et al., 2016). Increased expression of MMP13 has been reported in patients with spinal tuberculosis (Yang et al., 2019).

Apoptosis induced by ESAT-6 has been found to occur mainly through intrinsic pathways with elevated levels of cleaved caspase-9 and -3 proteins. Lower and higher expression of CASP9 has been reported in LC and PD patients, respectively (Ercan et al., 2019). It has been found that variants of IL1B were associated with latent tuberculosis infection, whereas variants of IL6 and TNFα variants were associated with pulmonary tuberculosis (Wu et al., 2018). IL-6 has been found associated with RA (Choy and Calabrese, 2018). Patients with Parkinson’s disease contain. Elevated levels of many pro-inflammatory cytokines such as IL-6, TNF, IL-1β, and IFNγ have been found elevated in PD (Sliter et al., 2018). ApoE deficiency was found associated with delayed adaptive immunity against TB (Martens et al., 2008). Increased expression of PD1 has been reported in patients with HIV and latent tuberculosis infection, leading to inhibition of IL-17, IL-22, and IL-23R activity towards CFP-10 and ESAT-6 *Mycobacterium tuberculosis* antigens (Devalraju et al., 2018). IL-22 has also been found associated with the progression of bone erosions (Kragstrup et al., 2018). Higher expression of IL22R1 has been reported in patients having *KRAS*-mutant lung adenocarcinoma (Khosravi et al., 2018). Rosas-Taraco et al. reported higher expression of CCR5 in pulmonary tuberculosis (Rosas-Taraco et al., 2006). The expression pattern of CCR5 also has been found associated with the development of diabetic nephropathy (Yahya et al., 2019). CCR5 is also a key gene in RA involved in recruiting inflammatory cells into the inflamed synovial tissue (Cai et al., 2019). IL-23 and IL23A have been reported associated with disease severity of type-2 DM and progression of RA (Eirís et al., 2014; Wendling et al., 2015). A recent study has shown the secretion of IL-23 by lung adenoma cells is associated with the generation of an inflammatory and immune-suppressed stroma (Lim et al., 2020). Gene polymorphisms modulating HLA Class I and II antigens are considered the risk factors of several diseases, including TB, DM, CVD, PD, and LC. HLA-A, HLA-B, HLA-DRB1, HLA-DQA1, and HLA-DQB1 were typed in two Manitoba First Nation indigenous groups to identify and compare the frequency of gene polymorphisms that may influence susceptibility or resistance to TB (Larcombe et al., 2017). The HLA-class II has been found associated with Type 1A DM(Sugihara et al., 2012). The HLA class 1 and 2 alleles were found associated with RA (Chan et al., 1994). HLA-A and HLAB antigens have been reported in patients with idiopathic PD (Marttila et al., 1981). HLA-A or HLA-B/C was found associated with up to 75% of LC cases (Talebian Yazdi et al., 2016).

The above-discussed literature showed that the majority of these key genes/regulators are associated with diverse processes and pathways, justifying their biological significance. The current study showed that these key genes/regulators are associated with TB and overlapping NCDs, namely DM, CVD, RA, PD, and LC. The finding of the current study, as well as from other studies, are providing us an insight into the overall molecular picture of these overlapping diseases and considering the fact that the TB and these NCDs are somewhere co-exist with each other at the gene level. This enables us to re-devise present strategies by looking at the collective effect of drugs on the genes commonly associated with TB and other overlapping NCDs; drug combinations could enhance the potency of a few drugs, given a synergistic effect and could also give better outcomes.

Further, there is little information on how TB treatment avoids getting an NCD or the value in selecting a drug if a patient gets an NCD. However, we have identified a total of 1975 drugs that show drug-target interactions. Among those interactions, many interactions are well known or previously reported in literature like many genes that co-exist in TB and NCDs are basically some cytokines (IL-1, TNF-α, IL-6, IL-12, and CXCL8. Etc.) and chemokines (CXCR4, CXCL8, CXCL10, CCL2, and CCL5) that are used as a target by a number of drugs including *Ibuprofen*, *Aspirin*, *Etoricoxib*, *Meloxicam*, *Celecoxib*, *Vitamin A*, *Cholecalciferol (* Supplementary Material S3 *)*. But many drugs show a direct interaction with our target proteins but no literature evidence available to support the interaction. So, additional research is needed to better understand the drug-target interaction and providing new research routes regarding a novel application of drugs not yet investigated in the specific context of TB and NCDs.

The study of proteins that interact with known disease-associated gene products in the human interactome and their subnetworks has enhanced our knowledge of disease mechanisms, including but are not limited to NCDs. Identification of previously unknown and shared mechanisms between different diseases has become possible with this network-based approach and may guide therapeutic strategies in the future and open a new horizon for more personalized treatment, drug-repurposing opportunities and uncover several side effects of unrelated drugs for TB and other complex diseases.

Finally, we acknowledge several potential limitations. First, although we integrated data from multiple sources to build the interactome and the drug-target network that relied on host gene/protein/disease datasets, their quality and literature bias may influence the performance and the results of network analysis. Second, our method can only be applied to diseases with well-characterized genetic information and may not be applicable for diseases that lack such information, such as rare diseases (i.e., cerebral palsy or mental conditions). Potential literature bias of disease-associated genes and the human interactome may also influence our findings.

## Conclusion

This study attempted to create a robust workflow taking TB and its overlapping NCDs into consideration and emphasize the need for an hour to re-think and re-devise therapies and therapeutic management. The findings of the current study also provide us an opportunity to focus on the untouched aspects of any disease, in particular with their distant related gene-sets (genes co-existence with other diseases)], and to work in synergy to have a collective physiological effect on one’s pathological phenotype. This study identified 86 target genes that co-exist in TB and NCDs. Targeting these targets using drugs combination or drug repurposing approaches will improve the clinical conditions in comorbidity, enhance the potency of a few drugs, and give a synergistic effect with better outcomes. TB and NCDs co-existence also creates opportunities for improved diagnosis and management of both. The existence of NCDs may indicate the need to actively TB screening for early TB detection. Similarly, diagnosis of TB should alert experts to actively screen for common non-communicable comorbidities, which may otherwise go undiagnosed. However, experimental validation of this study will be required to support further assessments of potential clinical application.

## Data Availability

The original contributions presented in the study are included in the article/Supplementary Material, further inquiries can be directed to the corresponding author.
