# Supplementary material for: An Integrative Network Approach to Identify Common Genes for the Therapeutics in Tuberculosis and Its Overlapping Non-Communicable Diseases
Source: Front Pharmacol. 2022 Jan 27;12:770762. doi: 10.3389/fphar.2021.770762 (PMC8829040; doi:10.3389/fphar.2021.770762)
Supplement: Supplementary file 1 [file DataSheet3.pdf]

33 High-scoring Significant Modules

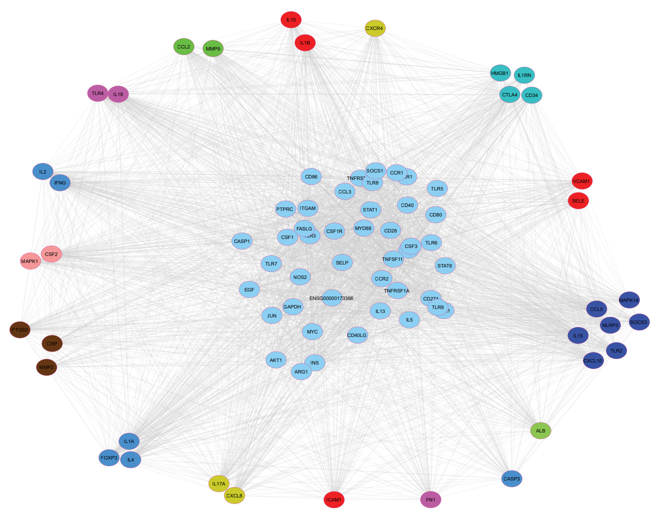

Module 1.1

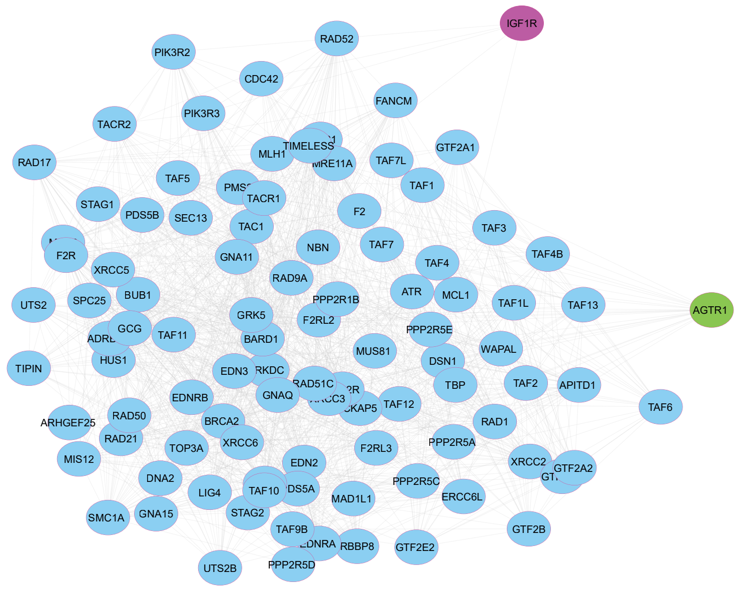

Module 1.2

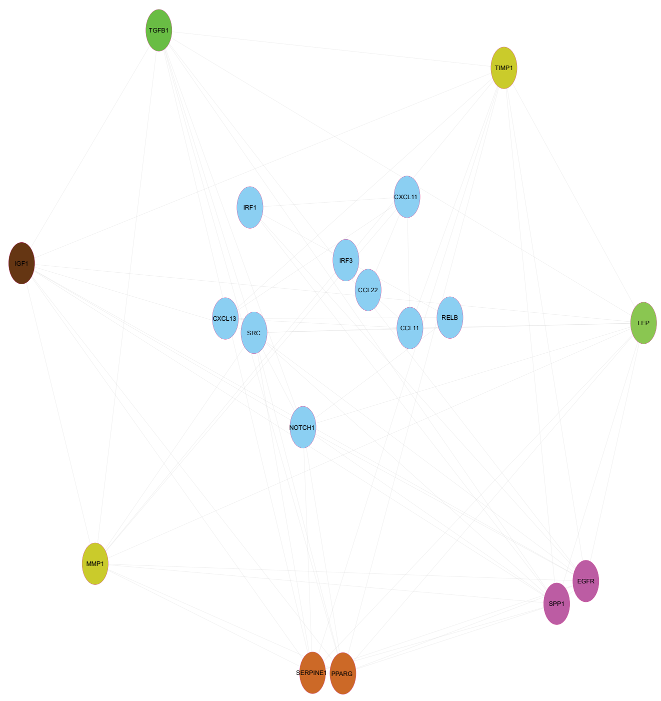

Module 1.3

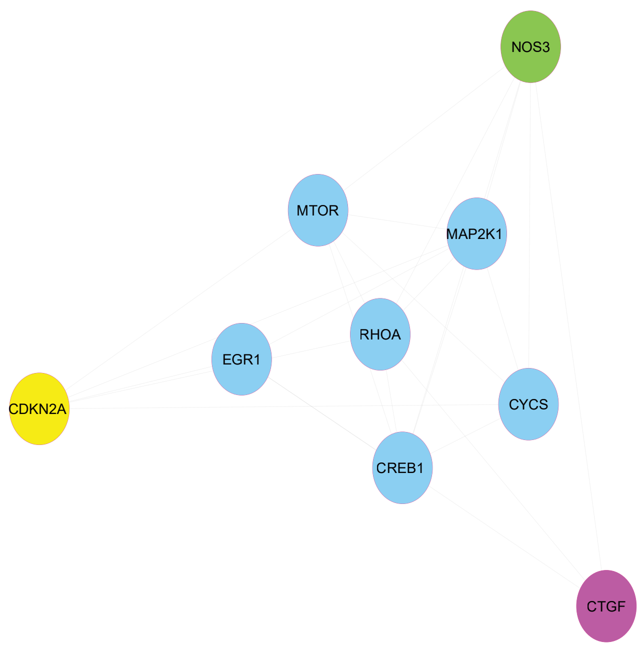

Module 1.4

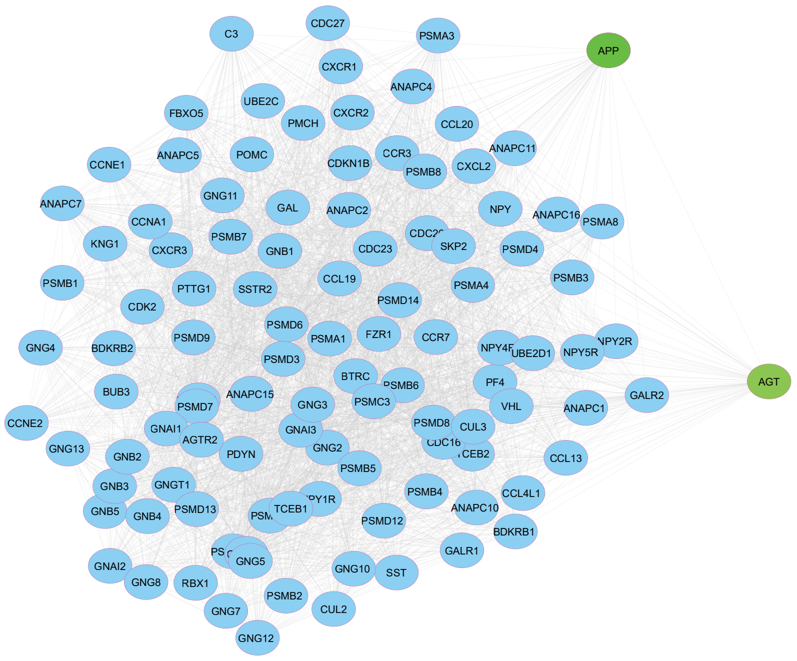

Module 2.1

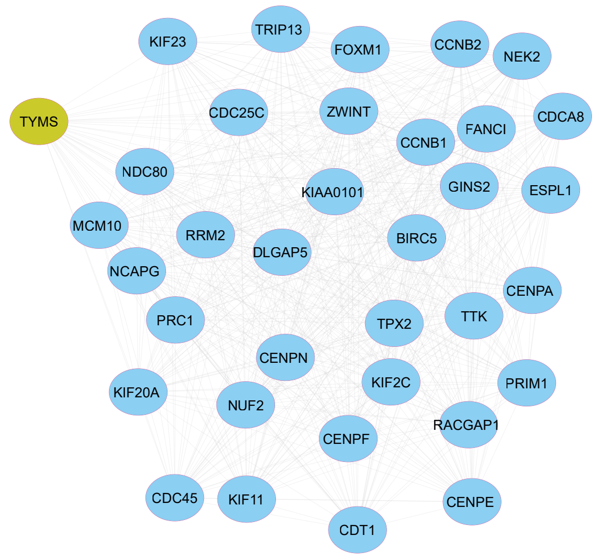

Module 2.2

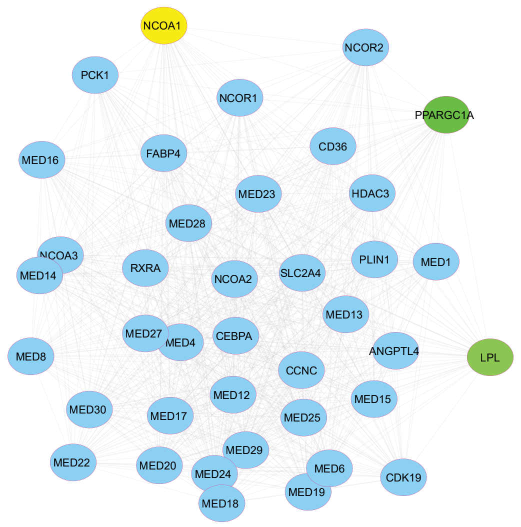

Module 3.1

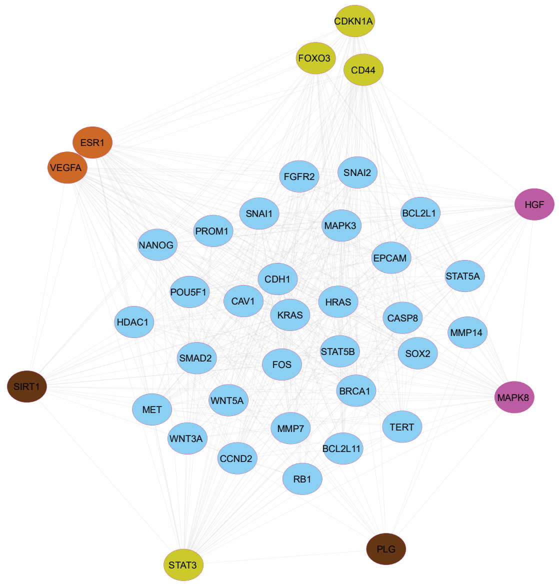

Module 3.2.1

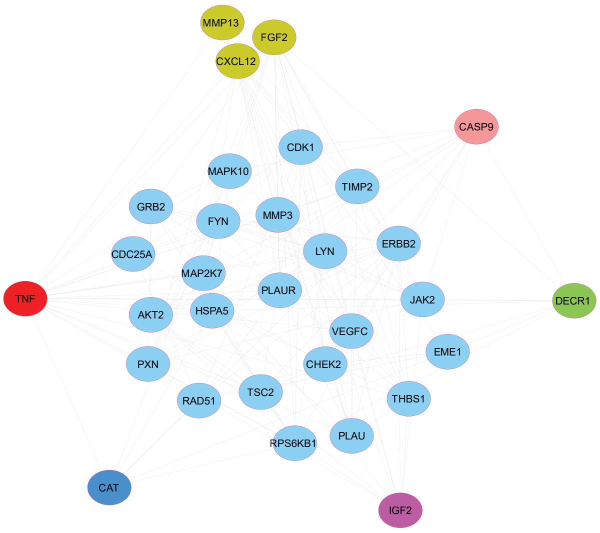

Module 3.2.2

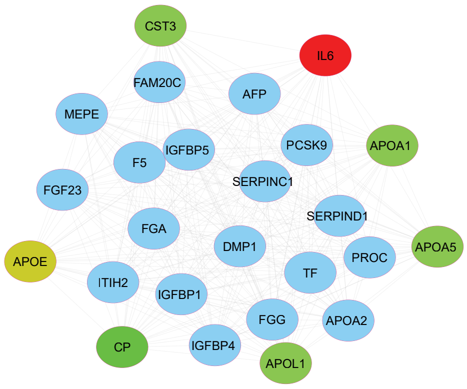

Module 3.3.1

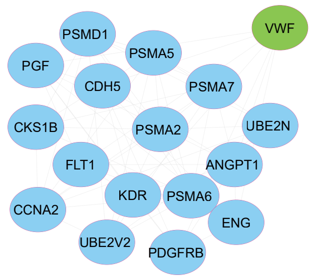

Module 3.4.1

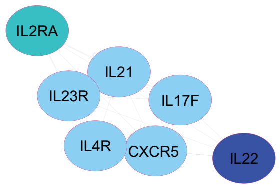

Module 3.4.2

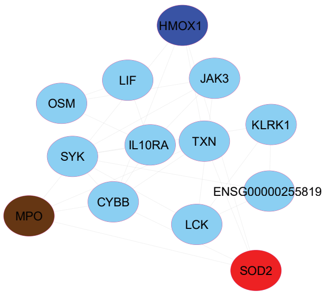

Module 3.4.3

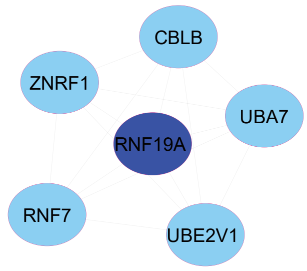

Module 3.5.1

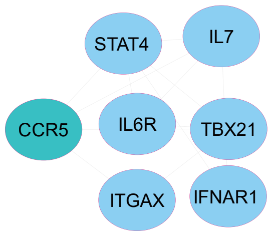

Module 3.5.2

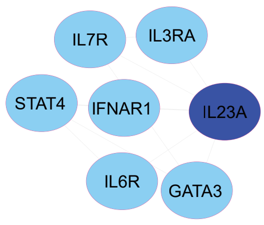

Module 3.5.3

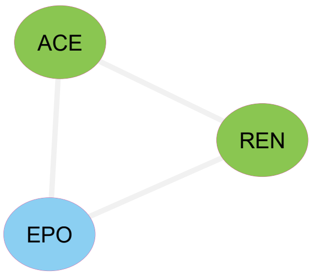

Module 3.6

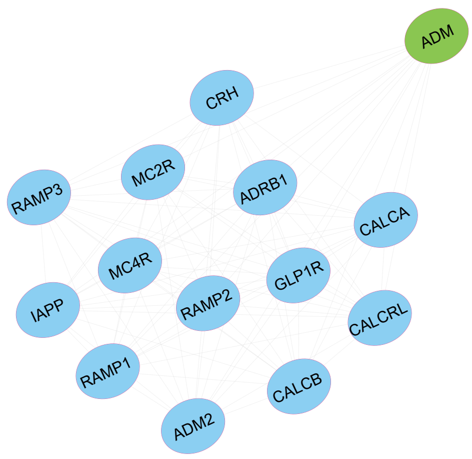

Module 4.1

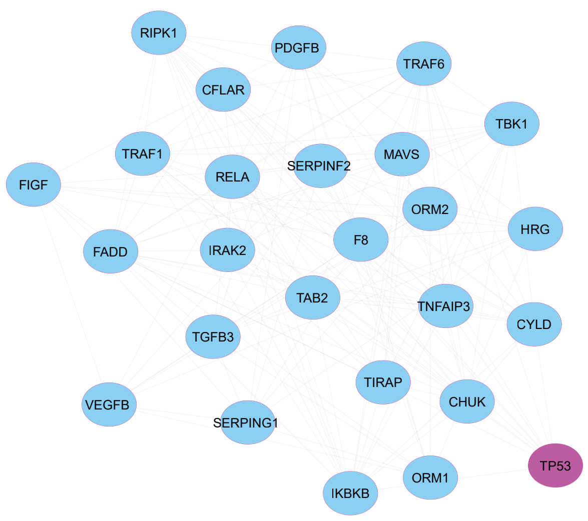

Module 4.2

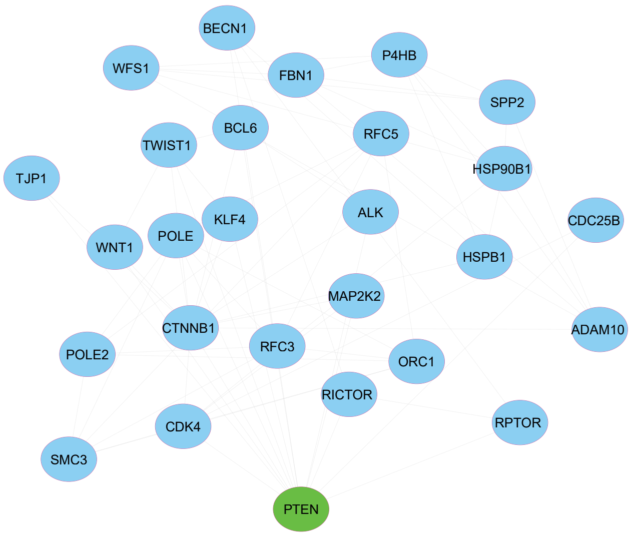

Module 4.3

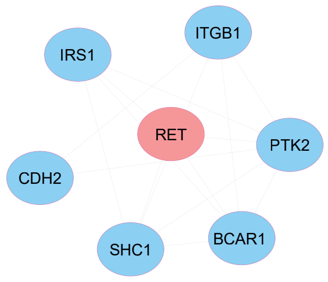

Module 4.4

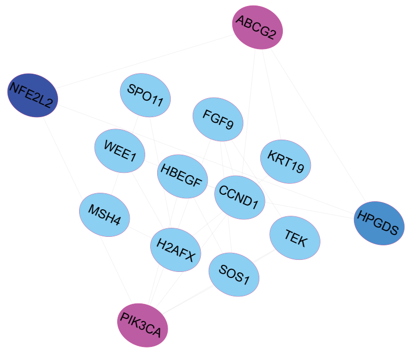

Module 4.5

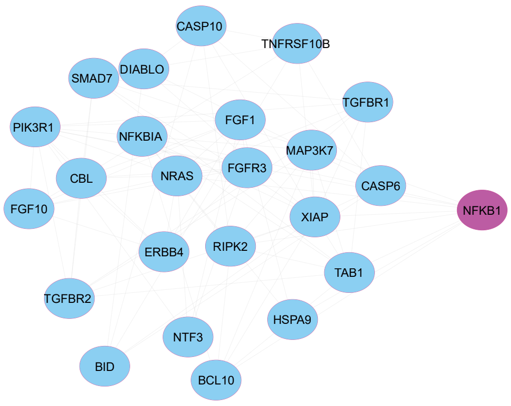

Module 5.1

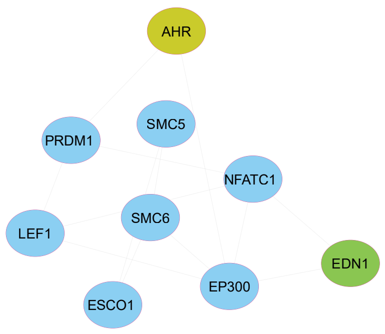

Module 5.2

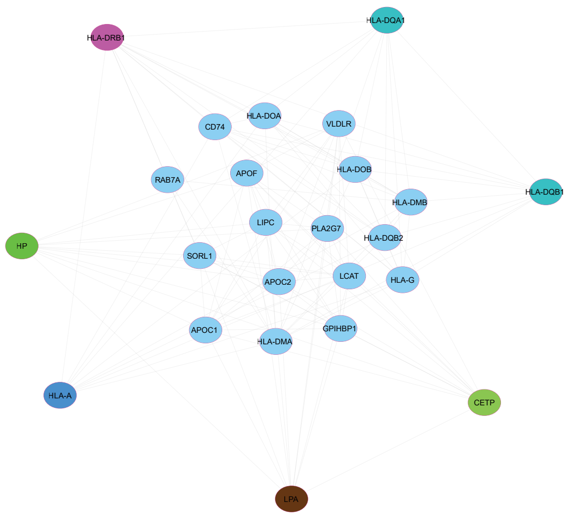

Module 6.1

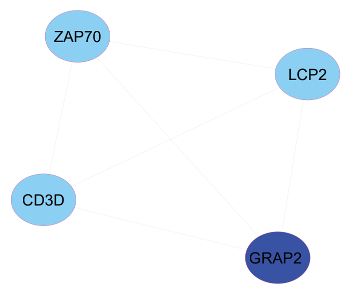

Module 6.2

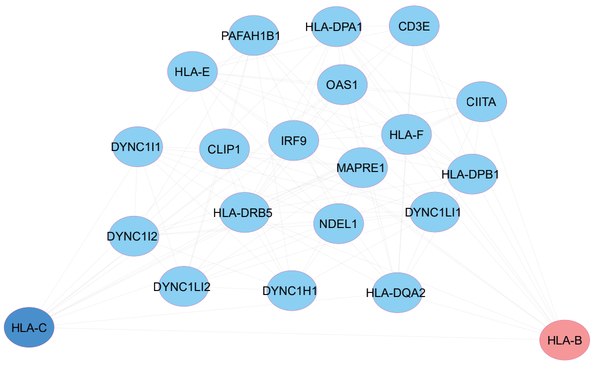

Module 7.1

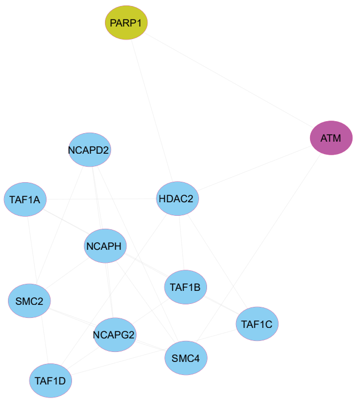

Module 7.2

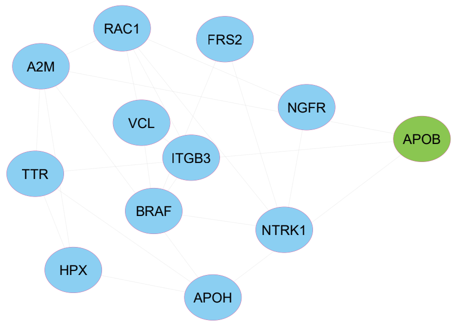

Module 7.3

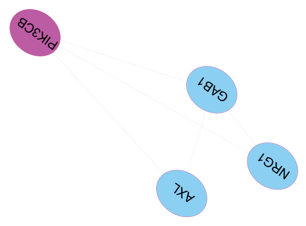

Module 7.4

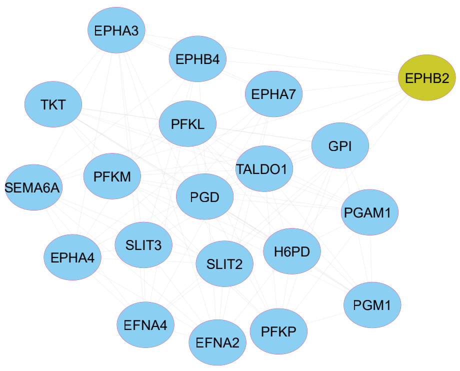

Module 8.1

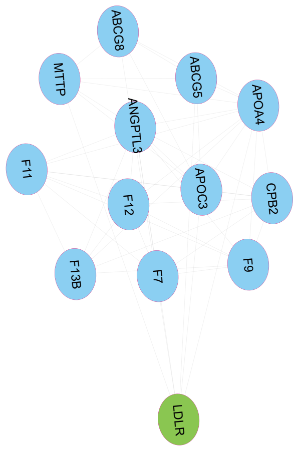

Module 8.2

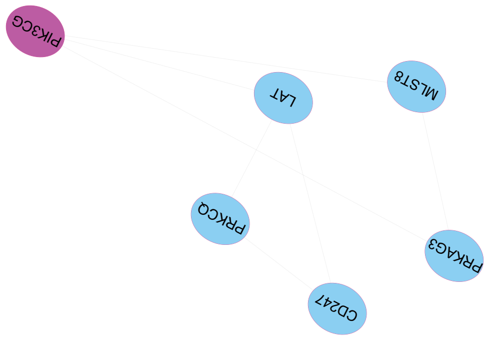

Module 8.3
