# Supplementary material for: An Integrative Network Approach to Identify Common Genes for the Therapeutics in Tuberculosis and Its Overlapping Non-Communicable Diseases
Source: Front Pharmacol. 2022 Jan 27;12:770762. doi: 10.3389/fphar.2021.770762 (PMC8829040; doi:10.3389/fphar.2021.770762)
Supplement: Supplementary file 2 [file DataSheet1.docx]

Suppli__Table 1: List of Drugs interacted with genes from all the 13 modules.

| CXCR4 | Framycetin, Rolofylline, Methionine Sulfoxide Chembl452864, Chembl460491, Plerixafor, Phenprocoumon, Bkt140, Ulocuplumab, Msx-122, Burixafor, Ctce-9908, Chembl1088913, Pol6326 and Chembl518924 |
| --- | --- |
| IL15 | Amg-714, Megestrol Acetate, Levodopa, Cyclosporine, Edaravone, Mycophenolate Mofetil, Phorbol Myristate Acetate, Sirolimus and |
| IL4 | Pascolizumab |
| TLR4 | Eritoran Tetrasodium, Chembl225157, Ritonavir, Chembl1232858, Saquinavir, Alcohol, Golotimod, Folic Acid, Eritoran, Infliximab, Nelfinavir, Dodecanoate and Myristic Acid |
| IL10 | Sch-708980, Tretinoin, Acyclovir, Tylosin, Mesalamine, Fluticasone Propionate, Ibudilast, Rofecoxib, Amoxicillin, Rabeprazole, Zidovudine, Clarithromycin, Lisofylline and Sirolimus |
| IFNG | Fontolizumab, Fumaric Acid, Melatonin, Melphalan, Apremilast, Olsalazine, Trastuzumab, Doxifluridine, Bleomycin, Amitriptyline, Glucosamine, Prednisone, Tretinoin, Interferon Alfa-2b, Ganciclovir, Cyclophosphamide, Ursodiol, Interleukin 1beta, Suramin, Theophylline, Pefloxacin, Methylprednisolone, Thrombin, Ibuprofen and Amikacin |
| MMP9 | Marimastat, Captopril, Glucosamine, Andecaliximab, Cgs-27023a, Ilomastat, Uridine Diphosphate Glucose, Prinomastat, Celecoxib, Bevacizumab, Incyclinide, Methyldopa, Nifedipine, |
| PTGS2 | Naproxen Etemesil, Valdecoxib, Celecoxib, Etodolac, Oxaprozin, Bromfenac Sodium, Meloxicam, Icosapent, Aminosalicylic Acid, Mesalamine, Indomethacin, Nabumetone, Tenoxicam, Lenalidomide, Rofecoxib, Piroxicam, Sulindac, Mefenamic Acid, Naproxen, Sulfasalazine, Phenylbutazone, Carprofen, Diflunisal, Suprofen, Salicylic Acid, Aspirin, Bromfenac, Ketoprofen, Balsalazide Disodium, Thalidomide, Lumiracoxib, Magnesium Salicylate, Salsalate, Ginseng, Asian, Antrafenine, Antipyrine, Tiaprofenic Acid, Etoricoxib, Resveratrol, Niflumic Acid, Nimesulide, Lornoxicam, Nepafenac, Parecoxib, Pomalidomide, Diclofenac, Naproxcinod, Apricoxib, Polmacoxib, Aminosalicylate Potassium, Flurbiprofen, Ketorolac Tromethamine, Olsalazine Sodium, Tolmetin, Chembl68211, Chembl364804, Ibuprofen, Arundic Acid, Bismuth Subsalicylate, Ibuprofen Sodium, Acetaminophen, Naproxen Sodium, Diclofenac Epolamine, Diclofenac Sodium, Oxyphenbutazone, Flurbiprofen Sodium, Tolmetin Sodium, Diclofenac Potassium, Aminosalicylate Sodium, Indomethacin Sodium, Oxaprozin Potassium, Ibuprofen Lysine, Fenoprofen Calcium, Meclofenamate Sodium, Parecoxib Sodium, Firocoxib, Oxaliplatin, Hydroxychloroquine, Meclofenamic Acid, Dersalazine, Phentermine Hydrochloride, Sulindac Sulfide, Fenoprofen, Ketorolac, Cimicoxib, Cyclosporine, Flufenamic Acid, Chembl374385, Dihomo-Gamma-Linolenic Acid, Licofelone, Icosapent Ethyl, Raloxifene, Reserpine, Capecitabine and Balsalazid, |
| CD34 | Prednisolone, Quercetin and Puromycin, |
| CASP3 | Chembl417149, Chembl586058, Emricasan, Idronoxil, Chembl293034, Celecoxib, Chembl310554, Paclitaxel and Rizatriptan, |
| FOXP3 | Epirubicin |
| CRP | Chembl406845 and Phosphocholine, |
| HMGB1 | Quinacrine Dihydrochloride, Chembl173373, Sodium Chloride, Hexamethylenebisacetamide, Sulfuric Acid, Itraconazole, Prednisolone, Epigalocatechin Gallate, Urea, Ampicillin, Chloramphenicol, Antibiotic and Volanesorsen Sodium, |
| ICAM1 | Enlimomab Pegol, Bi-505, Alicaforsen, Natalizumab, Hyaluronic Acid, and Lifitegrast, |
| MAPK1 | Isoprenaline, Arsenic Trioxide, Chir-99021, Ulixertinib, Gdc-0994, Mk-8353, Chembl1229592, Bumetanide, Benzoquinone, Refametinib, Medronic Acid, Erlotinib, Sb-203580, Ether, Chembl369507, Gonadotropin, , Chorionic, Turpentine, Chembl387385, Levodopa, Hypothemycin, Ursodiol, Chembl259551, Rebamipide, Doxifluridine, Irofulven, Chembl219841, Uracil, Sb-220025 and Olomoucine, |
| MAPK14 | Pf-03715455, Chembl1236539, Acumapimod, Azd-6703, Bms-582949, Propoxyphene, Dilmapimod, Doramapimod, Losmapimod, Chembl1951415, Ph-797804, Chembl306380, Ralimetinib, Sb-202190, Sb-203580, Sb-220025, Chembl313417, Tak-715, Talmapimod, Vx-702, Vx-745, Gsk-610677, Leo-15520, R-1487 (Chembl1766582), Ro-3201195, Rwj-67657, Sc-80036, Scio-323, Sd-0006, Ta-5493, Arry-797, Semapimod, Pg-760564, Ps-516895, Fx-005, Amg-548, Ave-9940, Sb-85635, Kc-706, Pamapimod, Pexmetinib, Ly-3007113, Azd-7624, Chembl305178, Hydrogen Peroxide, Chembl237127, 2-Chlorophenol, Chembl492265, Morphine, Resveratrol, Chembl193264, Chembl379760, Haloperidol, Chembl191598, Doxycycline, 2-Methoxyestradiol, Chembl478649, Chembl273158, Chembl96741, Ravidasvir, Chembl195393, Chembl590753, Chembl85860, Chembl94417, Chembl380373, Chembl195450, Doxorubicin, Vasopressin, Epoetin Beta, Chembl194009, Chembl494072, Chembl194322, Ralimetinib Mesylate, Sulindac Sulfide, Chembl87277, Chembl193156, Chembl564912, Chembl252128, Chembl29641, Chembl199237, Chembl559401, Chembl425616, Chembl197277, Phorbol Myristate Acetate, Methoxsalen, Lithium, Chembl522387, Chembl258202, Chembl410668, Gw607117x, Chembl365776, Pirfenidone, Chembl371491, Chembl522579 and Chembl257341, |
| VCAM1 | Carvedilol, Mercaptopurine, Dexamethasone, Phorbol Myristate Acetate, Succinobucol, Troglitazone, Cyclosporine |
| IL1RN | Rilonacept, Diacerein and Methotrexate, |
| CSF2 | Kb-003, Mor-103, Namilumab, Aclarubicin, Alcohol, Perfosfamide, Lactulose Hydrate, Famciclovir, Ticarcillin, Etanercept, Diaziquone, Mechlorethamine, Cytarabine, Omeprazole, Streptozotocin, Nordihydroguaiaretic Acid, Ditiocarb, Methimazole, Vinorelbin Ditartrate, Indinavir, Epigalocatechin Gallate, Mycophenolic Acid, Vinblastine, Sodium Salicylate, Cilostazol, Amikacin, Mt-203, Stavudine, Regramostim, Temozolomide, Roquinimex, Cetirizine, Interferon Alfa-2b, Beclomethasone Dipropionate, Isoproterenol, Miltefosine, Fluticasone Propionate, Bcg Vaccine, Trifluoperazine, Tetanus Toxoid, Procarbazine, Staurosporine, Mycophenolate Mofetil, Idarubicin and Terfenadine, |
| IL17A | Secukinumab, Ixekizumab, Chembl197194, Erythromycin, Tetracycline, Proxyphylline, Simvastatin, Mannitol, Sirolimus, Gentamicin, |
| FN1 | OCRIPLASMIN, AS-1409, L19tnfa, L19SIP, L19IL2, |
| NLRP3 | Anakinra |
| IL2 | Ticarcillin, Pentamidine Isethionate, Halofuginone, Chembl429852, Cyclosporine, Bromocriptine, Tropisetron, Diethylstilbestrol Diphosphate, Prochlorperazine, Interferon Beta-1b, Tylosin, Targinine, Imipenem, Cefepime, Acetaminophen, Pyrimethamine, Perfosfamide, Pravastatin, Quinine, Metoclopramide, Itraconazole, Midostaurin, Pefloxacin, Calcitriol, Trichostatin, Chembl106187, Roxithromycin, Beclomethasone, Clozapine, Methotrexate, Glyburide, Prasterone, Edaravone, Gentamicin, Aziridine, Ofloxacin, Garlic, Gonadotropin, Chorionic, Apremilast, Girentuximab, Amikacin Sulfate, Didemnin B, |
| CXCL8 | Rivanicline, Abx-Il8, Humax-Il8, Alprazolam, Troglitazone, Ionomycin, Phorbol Myristate Acetate, Hydroquinone, Medroxyprogesterone Acetate, Cetuximab, Aluminum Hydroxide, Omeprazole, Pamidronic Acid, Pentoxifylline, Danazol, Aspirin, Naproxen, Cidofovir, Talc, Tretinoin, Terfenadine, Tocopherol Acetate, Lansoprazole, Dipyridamole, Methimazole, Methylene Blue, Cyclophosphamide, Fenretinide, Acetaminophen, Chembl262489, Paclitaxel, Fentanyl, Ceftriaxone, Leflunomide, Midazolam, Rutin, Bevacizumab, Foscarnet, Chembl1213492, Verapamil And Clarithromycin |
| CXCL10 | Ni-0801, Regramostim, Oxaliplatin, Zidovudine, Eldelumab, Gonadotropin, Chorionic, Atorvastatin, Methylprednisolone, Testosterone, Antibiotic, Atropine, Stavudine, Ritonavir, |
| IL1B | Canakinumab, Rilonacept, Gallium Nitrate, Amg-108, Ibudilast, Vx-702, Celastrol, Nicardipine, Acitretin, Pentamidine, Resveratrol, Ofloxacin, Raloxifene, Mafosfamide, Cytarabine, Hydroquinone, Belnacasan, Melatonin, Nimustine Hydrochloride, Talmapimod, Verapamil, Pentoxifylline, Gevokizumab, Thyroglobulin, Cefaclor, Phorbol Myristate Acetate, Medronic Acid, Diacerein, Clodronate Disodium, Erythromycin, Lansoprazole, Fluticasone Propionate, Sodium Beta-Nicotinamide Adenine Dinucleotide Phosphate, Hydrocortisone, Lithium, Beclomethasone Dipropionate And Etiprednol Dicloacetate |
| IL1A | Rilonacept, Ra-18c3, Hydroxychloroquine, Amg-108, Mitomycin, |
| ALB | Gadobenate Dimeglumine, Ebselen, Sodium Lauryl Sulfate, Bismuth Subsalicylate, Iodipamide, Rifampicin, Cefotaxime, Naproxen And Raltitrexed. |
| CCL2 | Danazol, Chembl134074 And Carlumab |
| IL18 | Mycophenolic Acid, Dextran Sulfate Sodium, Thyroxine, Zidovudine, Tacrolimus And Colchicine, |
| MMP2 | Marimastat, Captopril, Chembl181244, Cgs-27023a, Ilomastat, Tanomastat, Tiludronic Acid, Incyclinide, Prinomastat, Rebimastat, Batimastat, Solimastat, Paclitaxel, Resveratrol, Pravastatin, Streptozotocin, Filgrastim, Letrozole, Halofuginone, Simvastatin, Perfosfamide, Chembl1233506, Chondroitin Sulfate, Cyclosporine, Zoledronic Acid, Ramipril, Vinblastine, Bevacizumab, Etretinate, Chlorhexidine, |
| SELE | Carvedilol, Bimosiamose, Rivipansel, Chembl1234621 |
| CCL5 | Fluticasone Propionate |
| CTLA4 | Ipilimumab, Tremelimumab, Wortmannin, Sirolimus, Alcohol, Methimazole, Prednisone, Thalidomide, Atpgammas, Rizatriptan, Melphalan, Atezolizumab, Phorbol Myristate Acetate, Bleomycin (Chembl3039590), Antibiotic, |
| TLR2 | Golotimod |
| PPARG | Ave0847, Imiglitazar, Tesaglitazar, Pioglitazone Hydrochloride, Rosiglitazone Maleate, Mesalamine, Db959, Indeglitazar, Etalocib, Aleglitazar, Muraglitazar, Icosapent, Troglitazone, Indomethacin, Rosiglitazone, Nateglinide, Sulfasalazine, Repaglinide, Telmisartan, Balsalazide Disodium, Glipizide, Pioglitazone, Mitiglinide, Bezafibrate, Edaglitazone, Tagitinin A, Tirotundin, Chembl88496, Ciglitazone, Chembl434063, Chembl410478, Gw7845, Gsk-9578, L-165461, Chembl23296, L-796449, Linoleic Acid, Ly-510929, Chembl24458, Netoglitazone, Ragaglitazar, Reglitazar, Chembl1946409, Bardoxolone, Farglitazar, Chembl149394, Chembl181937, Chembl179330, Bexarotene, Bardoxolone Methyl, Diclofenac, Resveratrol, Chembl510698, Chembl375270, Clx-0921, Balaglitazone, Rivoglitazone, Efatutazone, Orantinib, Sodelglitazar, Naveglitazar, Oms405, Mbx-2044, Int131, Atx08-001, Efatutazone Hydrochloride, Arhalofenate, Olsalazine Sodium, Mk-0533, Genistein, Metaglidasen, Chembl364748, Treprostinil, Chembl201880, Elafibranor, Icosapent Ethyl, Olanzapine, Chembl146624, Celecoxib, Chembl86658, Chembl1210221, Zoledronic Acid, Chembl191060, Balsalazid, Chembl1204498, Chembl1230670, Chembl191275, Chembl378160, Gw501516, Chembl200495, Chembl451721, |
| MMP1 | Marimastat, Cgs-27023a, Cipemastat, Ilomastat, Doxycycline Hydrate, Doxycycline Hyclate, Doxycycline Calcium, Solimastat, Batimastat, Prinomastat, Rebimastat, Collagenase Clostridium Histolyticum, Pentosan Polysulfate Sodium, Hydrocortisone, Sirolimus, Vitamin E, Lamivudine, Leflunomide, Interferon Beta, Triamcinolone, Chembl92608, Medroxyprogesterone Acetate, Ribavirin, Leuprolide Acetate, Chembl11306, Chembl45483, |
| SPP1 | Ask-8007, Tacrolimus, Zoledronic Acid, Tretinoin, Chondroitin Sulfate, Sodium Butyrate, Calcitonin, Wortmannin, Phorbol Myristate Acetate, Gentamicin |
| TGFB1 | Hyaluronidase (Human Recombinant), Metelimumab, Fresolimumab, Ly-2382770, Atenolol, Nicotine, Hydrocortisone, Etoposide, Glatiramer Acetate, Etretinate, Inositol, Amifostine, Testosterone, Interferon Alfa-2b, Gallium Nitrate, Pioglitazone, Fenretinide, Tretinoin, Pirfenidone, Triamcinolone, Vitamin E, Hydrogen Peroxide, Toremifene, Isoflavone, Estradiol, Melatonin, Isotretinoin, Streptozotocin, Cladribine, Gossypol, Emodin, Verapamil, Ramipril, Tamoxifen, Genistein, Idoxuridine, Doxorubicin |
| EGFR | Cetuximab, Gefitinib, Erlotinib, Panitumumab, Necitumumab, Acalabrutinib, Aee-788, Afatinib, Tyrphostin Ag-1478, Chembl56543, Bgb-283, Ac-480, Bms-690514, Chembl174426, Brigatinib, Canertinib, Felypressin, Cudc-101, Dacomitinib, Dovitinib, Chembl387187, Falnidamol, Ibrutinib, Icotinib, Lapatinib, Naquotinib, Neratinib, Olmutinib, Cholecalciferol, Orantinib, Osimertinib, Chembl53753, Pelitinib, Chembl1951415, Pki-166, Poziotinib, Chembl306380, Chembl1081312, Rociletinib, Sapitinib, Tesevatinib, Vandetanib, Chembl1229592, Chembl2141478, Duligotuzumab, Futuximab, Nimotuzumab, Imgatuzumab, Momelotinib, Zalutumumab, Erlotinib Hydrochloride, Lapatinib Ditosylate, Depatuxizumab, S-222611, Sb-243213, Azd-4769, Nitroglycerin, Nepidermin, Mubritinib, Afatinib Dimaleate, Osimertinib Mesylate, Allitinib, Cep-32496, Hm-61713, Epitinib, Mp-412, Pd-0166285 (Chembl3545196), Tak-285, Pyrotinib, Varlitinib, Egf816, Canertinib Dihydrochloride, Dacomitinib Hydrate, Simotinib, Mab-425, Rg-7160, Puquitinib, Theliatinib, Jnj-26483327, Matuzumab, Azd-4547, Potassium Chloride, Ponatinib, Dactolisib, Ganetespib, Atezolizumab, Chembl1231206, Paclitaxel, Golvatinib, Dasatinib, Chembl1765740, Erismodegib, Selumetinib, Vemurafenib, Trastuzumab, Linsitinib, Encorafenib, Alvocidib, Pha-665752, Jnj-42756493, Capreomycine, Decitabine, Mk-2206, Ign-311, Procaterol, Savolitinib, Dabrafenib, Nvp-Auy922, Geldanamycin, Imatinib, Insm-18, Trametinib, Pimasertib, Mgcd-265, Alcohol, Regorafenib, Crizotinib, Everolimus, Mdx-447, Sorafenib, Pembrolizumab, Amlexanox, Lidocaine, Rindopepimut, Bevacizumab, Buparlisib, Irinotecan, Bosutinib, Gedatolisib, Pemetrexed (Chembl1201258), Capmatinib, Sirolimus, Amg-337, Chembl285063, Temozolomide, Temsirolimus, Sunitinib, Depatuxizumab Mafodotin, Saracatinib, Durvalumab, Chembl458997, Carboplatin, Bms-754807, Cabozantinib, Etoposide, Nivolumab, |
| SERPINE1 | Troglitazone, Aleplasinin, Fluoxetine, Hydrochlorothiazide, Alteplase, Drotrecogin Alfa (Activated), Levothyroxine, Epirubicin, Vasopressin, Urokinase, Cetrorelix, Regramostim, Anistreplase, Fibrinolysin, Reteplase, Dalteparin Sodium, Orlistat, Nimodipine, Defibrotide, Tenecteplase, Etretinate, Glycyrrhizin, Arsenic Trioxide, Uridine, Captopril, Estrogens, |
| LEP | Bromocriptine, Methadone, Gemfibrozil, Lovastatin, Troglitazone, Soybean Oil, Valproic Acid, Felodipine, Carvedilol, Estradiol Valerate, Folic Acid, Risperidone, Propylthiouracil, Olanzapine, Methimazole, Lamivudine, Mirtazapine, Clozapine, |
| IGF1 | Dusigitumab And Chembl1235854 |
| MAPK8 | Metaraminol, Hexane, Chembl210618, Sp-600125, Cc-401, Bentamapimod, Tanzisertib, Chembl382639, Vitamin E, Tretinoin, Gefitinib, Butylated Hydroxytoluene, Oxytocin, 2-Methoxyestradiol, Dextran Sulfate Sodium, Enalapril, Prazosin, Chembl207228, Bleomycin (Chembl3039590), Doxycycline, Chembl209740, Bexarotene, Chembl242237, Dexrazoxane, |
| VEGFA | Muparfostat (Chembl1615835), Aflibercept, Carvedilol, Vandetanib, Dalteparin Sodium, Bevasiranib Sodium, Conbercept, Brolucizumab, Pegaptanib Sodium, Ranibizumab, Bevacizumab, Tak-593, Semaxanib, Sorafenib Tosylate, Lithocholic Acid, Chembl313417, Juglone, Bevacizumab 111in, Tromethamine, Pentosan Polysulfate Sodium, Pegaptanib Octasodium, Sorafenib, Celecoxib, Pyroglutamic Acid, Chembl262489, Gliclazide, Phenytoin, Lenalidomide, Bevasiranib, Gentamicin, Cilostazol, Chembl88153, Fenofibrate, |
| CD44 | Hyaluronic Acid, Staurosporine, Interferon Gama-1b, Mycophenolic Acid, Gentamicin, Mometasone Furoate |
| ESR1 | Afimoxifene (Chembl489), Diarylpropionitrile, Diethylstilbestrol, Chlorotrianisene, Estrogens, Conjugated, Etonogestrel, Desogestrel, Levonorgestrel, Progesterone, Toremifene, Medroxyprogesterone Acetate, Estrone, Tamoxifen, Dienestrol, Fulvestrant, Norgestimate, Ethinyl Estradiol, Melatonin, Trilostane, Fluoxymesterone, Estramustine, Estriol, Prinaberel, Propylpyrazoletriol, Raloxifene, Chembl282489, Chembl188528, Lasofoxifene, Bazedoxifene, Clomiphene, Chembl201013, Cholecalciferol, Hexestrol, Chembl520107, Mestranol, Danazol, Allylestrenol, Prasterone, Estropipate, Quinestrol, Ospemifene, Tibolone, Estrogens, Conjugated Synthetic A, Synthetic Conjugated Estrogens, B, Estradiol, Mitotane, Sr16234 (Chembl3545210), Fispemifene, Ly2245461, Idoxifene, Gtx-758, Afimoxifene (Chembl10041), Droloxifene, Acolbifene, Tamoxifen Citrate, Estradiol Valerate, Clomiphene Citrate, Estradiol Cypionate, Estrogens, Esterified, Diethylstilbestrol Diphosphate, Toremifene Citrate, Bazedoxifene Acetate, Chf4227, Gdc-0810, Estradiol Acetate (Chembl1200430), Polyestradiol Phosphate, Mk-6913, Chembl391910, Chembl180300, Dienogest, Rad1901, Chembl193676, Ethynodiol Diacetate, Ribociclib, Raloxifene Core, Chembl236718, 2-Amino-1-Methyl-6- Phenylimidazo[4,5- B]Pyridine, Endoxifen, Megestrol Acetate, Estrone Sodium Sulfate, Letrozole, Lapatinib, Carboquone, Iodine, Vintafolide, Custirsen, Chembl304552, Everolimus, Chembl181936, Pertuzumab, Estrogen, Chembl222501, Arzoxifene, Chembl180071, Genistein, Raloxifen, Trastuzumab, Abemaciclib, Erteberel, Exemestane, Sivifene, Palbociclib, Leflunomide, Chembl236086, Chembl184151, Chembl223026, Chembl180517, Norelgestromin, Anastrozole, Gonadorelin, Norgestrel, |
| STAT3 | Acitretin, Atiprimod, Bardoxolone Methyl, |
| SIRT1 | Chembl257991, Sodium Lauryl Sulfate, Chembl420311, Splitomicin, Resveratrol, |
| HGF | Rilotumumab, Ficlatuzumab, Methotrexate, Resveratrol, Gonadotropin, Chorionic, Wortmannin, Chembl262489, Sodium Heparin, Reserpine, Trastuzumab, Cabozantinib, Foretinib, Aspirin, Epigalocatechin Gallate, Thalidomide, Streptozotocin, |
| CDKN1A | Chembl3186227, Dicumarol, Paclitaxel, Melphalan, Deoxycytidine, Diaziquone, Acetaminophen, Dextran Sulfate Sodium, Nicotine, Arsenic Trioxide, Tipifarnib, Vinblastine, Cladribine, Carboplatin, Sodium Salicylate, Celecoxib, Sodium Phenylbutyrate, Epoetin Beta, Nimustine Hydrochloride, Depsipeptide, Fluorouracil, Irinotecan, Cyclosporine, Flutamide, |
| PLG | Tenecteplase, Streptokinase, Reteplase, Alteplase, Urokinase, Anistreplase, Tranexamic Acid, Aminocaproic Acid, Aprotinin, Chembl2016873, Defibrotide Sodium, Desmoteplase, Coumarin, Garlic, Amediplase, Phorbol Myristate Acetate, Inositol, Chembl35482, Prednisolone, Oxymetholone, Genistein, Soybean Oil, Norethindrone, Lepirudin, Danazol, Penicillin G Sodium, Nystatin, Fibrinolysin |
| DECR1 | Chembl1161862 And Chembl174539 |
| MMP13 | Doxycycline Hydrate Doxycycline Calcium Marimastat Chembl116757 Chembl222002 Prinomastat Uridine Diphosphate Glucose Chembl327002 Doxycycline Hyclate Chembl236573 Chembl514794 Chembl468900 Chembl511942 Chembl409536 Chembl1234479 Chembl93687 Chembl70176 Chembl440498 Chembl496942 3-Methylpyridine |
| TNF | Etanercept, Adalimumab, Infliximab, Thalidomide, Inamrinone, Golimumab, Certolizumab Pegol, Pomalidomide, Nafamostat, Talactoferrin Alfa, Urapidil, Placulumab, Delmitide, Chembl219629, Vadimezan, Pirfenidone, Ajulemic Acid, Pentoxifylline, Lenalidomide, Ortataxel, Lenercept, Nerelimomab, Afelimomab, Onercept, Ozoralizumab, Pegsunercept, Az-9773, Chloroquine, Risperidone, Apremilast, Lactulose Hydrate, Omeprazole, Glucosamine, Bupivacaine, Dersalazine, Phorbol Myristate Acetate, Carbamazepine, Celastrol, Midazolam, Procarbazine, Propylthiouracil, Timolol Maleate, Meropenem, Halofuginone, Didanosine, Folic Acid, Spironolactone, Methimazole, Pf-04236921, Beta-Lapachone, Clenbuterol, Vx-702, Talmapimod, Hydroxychloroquine, Pranlukast, Magnesium Sulfate, Chembl173373, Abacavir, Rabeprazole, Midostaurin, Miltefosine, Ibudilast, Rutin, Prazosin Hydrochloride, Pyridoxine, Penicillin G Sodium, Cefotaxime, Gentamicin, Glimepiride, Nordihydroguaiaretic Acid, Cromolyn Sodium, Fluocinolone Acetonide, Dusigitumab, |
| IGF2 | Dusigitumab, Chembl89363, Etoposide, Betamethasone, Cabazitaxel, Glutamine, Anastrozole, Fluoxetine, Gefitinib, Linsitinib, Antazoline Hydrochloride And Docetaxel. |
| CASP9 | Nivocasan, Emricasan And Celecoxib |
| CXCL12 | Tinzaparin Sodium |
| FGF2 | Sucralfate, Pentosan Polysulfate Sodium, Muparfostat (Chembl1615835), Chembl262489, Triamcinolone, Caffeine, Vincristine, Medronic Acid, Sirolimus, Indomethacin, Edaravone, Fulvestrant, Thyrotropin, Phenylephrine, Atorvastatin, Estradiol Dipropionate, Pyrazole, Aspirin, Rebamipide, Quizartinib And Famotidine. |
| CAT | Fomepizole |
| IL6 | Ginseng, Asian, Siltuximab, Sirukumab, Olokizumab, Elsilimomab, Pf-04236921, Ibudilast, Clazakizumab, Cdp-6038, Fentanyl, Saquinavir, Midostaurin, Dihydrospingosine, Gallium Nitrate, Nelfinavir, Gemfibrozil, Linezolid, Interferon Alfa-2b, Ifosfamide, Vx-702, Levofloxacin, Vitamin K, Metronidazole, Arsenic Trioxide, |
| APOA1 | Lamivudine, Glucagon, Testosterone, Tretinoin, Alcohol, Ritonavir, Furosemide, Apabetalone, Fenofibrate, |
| CST3 | Digoxin And Ribavirin |
| APOE | Cerivastatin, Troglitazone, Tretinoin, Ganciclovir, Triamcinolone, Albumin Human, Staurosporine, Ginkgo, Irbesartan, Vitamin E, Simvastatin, Lorazepam, Prednisone, Lutein, Buthionine Sulfoximine, Ritonavir, Soybean Oil, Gonadotropin, Chorionic, |
| IL2RA | Inolimomab, Denileukin Diftitox, Human Interleukin-2, Basiliximab, Daclizumab, Beclomethasone Dipropionate, Megestrol Acetate, Paclitaxel, Cladribine, L-778123, Cetirizine, Didanosine, Methimazole, Lentinan, Caffeine, Methylprednisolone, Gonadotropin, Chorionic, Mercaptopurine, Propylthiouracil, Verapamil, As-1409, Flutamide, Interferon Beta-1b, Theophylline, Staurosporine, Fluorouracil, Sodium Beta-Nicotinamide Adenine Dinucleotide Phosphate, Decitabine, Progesterone, Atorvastatin, Isotretinoin, Lmb-2, Thyroxine, Garlic, Dextran Sulfate Sodium, Bryostatin, |
| IL22 | Fezakinumab |
| HMOX1 | Biliverdin, Zinc Chloride, Sunitinib, Sorafenib, Chembl1161866, Formic Acid, Aspirin, |
| MPO | Cefdinir, Melatonin, Chembl310871, Loratadine, Trimethoprim, Tolmetin, Doxycycline, Tocopherol Acetate, Mesalamine, Diclofenac, Levocarnitine, Fludarabine, Octreotide, Lithium, Filgrastim, Theophylline, Tenecteplase, Nimesulide, Nomifensine, Bromoacetic Acid, Psoralen, Pyrazinamide, Dimethyl Sulfoxide, Fentanyl, Lidocaine, Amsalog, Sodium Beta-Nicotinamide Adenine Dinucleotide Phosphate, Flutamide, Methylene Blue, |
| SOD2 | Antibiotic And Cyclophosphamide |
| CCR5 | Guanidino Acetate, Guanine, Bicarbonate, Glycerin, Enoxaparin, Ancriviroc (Chembl2107311), Aplaviroc, Azd5672, Cenicriviroc, Chembl1196395, Maraviroc, Vicriviroc, Phenprocoumon, Pf-04634817, Incb-9471, Aplaviroc Hydrochloride, Vicriviroc Maleate, Chembl78535, Chembl207004, Pro-140, Chembl41275, Chembl518924, Glatiramer Acetate, |
| IL23A | Guselkumab, Briakinumab, Ustekinumab, Tildrakizumab, |
| LPA | Aminocaproic Acid |
| CETP | Anacetrapib, Dalcetrapib, Torcetrapib, Chembl67129, Evacetrapib, Cerivastatin, Tamoxifen, |
| HLA-DQB1 | Clavulanic Acid And Amoxicillin |
| HP | Pyridoxine, Estradiol, Streptozotocin, |
| HLA-DRB1 | Glatiramer Acetate, Lapatinib, Lym-1, Lapatinib, Interferon Beta-1b, Interferon Beta-1a, |
| HLA-A | Carbamazepine, Indole-3-Butyric Acid |
| HLA-B | Pazopanib, Carbamazepine, Oxcarbazepine, Phenytoin, Abacavir, Thalidomide, |
|  |  |
